# Abrupt and altered cell-type specific DNA methylation profiles in blood during acute HIV infection persists despite prompt initiation of ART

**DOI:** 10.1371/journal.ppat.1009785

**Published:** 2021-08-13

**Authors:** Michael J. Corley, Carlo Sacdalan, Alina P. S. Pang, Nitiya Chomchey, Nisakorn Ratnaratorn, Victor Valcour, Eugene Kroon, Kyu S. Cho, Andrew C. Belden, Donn Colby, Merlin Robb, Denise Hsu, Serena Spudich, Robert Paul, Sandhya Vasan, Lishomwa C. Ndhlovu, the SEARCH010/RV254 and SEARCH013/RV304 study groups

**Affiliations:** 1 Department of Medicine, Division of Infectious Diseases, Weill Cornell Medicine; New York, New York, United States of America; 2 Institute of HIV Research and Innovation; Bangkok, Thailand; 3 SEARCH, South East Asia Research Collaboration in HIV; Bangkok, Thailand; 4 Memory and Aging Center, University of California San Francisco; San Francisco, California, United States of America; 5 Missouri Institute of Mental Health University of Missouri; St. Louis, Missouri, United States of America; 6 Armed Forces Research Institute of Medical Sciences; Bangkok, Thailand; 7 Henry M. Jackson Foundation for the Advancement of Military Medicine; Bethesda, Maryland, United States of America; 8 Department of Neurology, Yale University; New Haven, Connecticut, United States of America; 9 US Military HIV Research Program; Silver Spring, Maryland, United States of America; Institut Cochin, INSERM U1016, FRANCE

## Abstract

HIV-1 disrupts the host epigenetic landscape with consequences for disease pathogenesis, viral persistence, and HIV-associated comorbidities. Here, we examined how soon after infection HIV-associated epigenetic changes may occur in blood and whether early initiation of antiretroviral therapy (ART) impacts epigenetic modifications. We profiled longitudinal genome-wide DNA methylation in monocytes and CD4^+^ T lymphocytes from 22 participants in the RV254/SEARCH010 acute HIV infection (AHI) cohort that diagnoses infection within weeks after estimated exposure and immediately initiates ART. We identified monocytes harbored 22,697 differentially methylated CpGs associated with AHI compared to 294 in CD4^+^ T lymphocytes. ART minimally restored less than 1% of these changes in monocytes and had no effect upon T cells. Monocyte DNA methylation patterns associated with viral load, CD4 count, CD4/CD8 ratio, and longitudinal clinical phenotypes. Our findings suggest HIV-1 rapidly embeds an epigenetic memory not mitigated by ART and support determining epigenetic signatures in precision HIV medicine.

**Trial Registration:**NCT00782808 and NCT00796146.

## Author summary

The epigenetic marker, DNA methylation, plays a key role regulating the immune system during host-pathogen interactions. Using cell-type specific DNA methylation profiling, we explored whether epigenetic changes occurred soon after HIV infection and following early treatment with anti-HIV drugs. Acute infection was associated with early DNA methylation changes in purified monocytes and CD4^+^ T cells isolated from blood. In monocytes, rapid anti-HIV treatment minimally restored DNA methylation changes associated with infection and unexpectedly had no impact in CD4^+^ T cells. DNA methylation patterns before treatment informed long term clinical outcomes including CD4^+^ T cell counts and favorable clinical phenotypes. These findings identify candidates for consideration in epigenome editing approaches in HIV prevention, treatment, and cure strategies.

## Introduction

Human immunodeficiency virus (HIV-1) infection is characterized by a rapid takeover of the host immune system and lasting impact on the immune system. Multiple studies provide evidence that HIV-induced cellular reprogramming occurs in host immune cells by altering epigenetic processes, including DNA modifications such as DNA methylation[1], chromatin landscape and accessibility[2,3], three-dimensional chromatin organization[4], and cell type-specific transcriptional programs[5]. Together, these studies suggest that HIV-1 targets the reshaping of the host epigenome to drive transcriptional changes related to dysfunctional innate and adaptive immune defenses, ultimately promoting immune evasion, viral replication, and viral persistence. However, *in vivo* knowledge about how early host epigenetic changes to immune cells occur during acute HIV-1 infection (AHI) and the impact of early initiation of combination antiretroviral therapy (ART) on the epigenome during AHI remains unclear.

Due to the critical role of the epigenetic marker DNA methylation as a stable and crucial transcriptional regulator of the immune system during host-pathogen interactions[6], DNA methylation is a well-studied epigenetic modification in HIV-1 infection. Human DNA methylation profiling studies of bulk host immune cells during chronic HIV-1 infection reveal epigenetic signatures of exposure, disease progression, advanced epigenetic aging, and HIV-1-related comorbidities[7–13]. Additionally, a recent report of untreated HIV-1 infected individuals stratified by high or low plasma viral loads found that host immune genes involved in HIV-1 viral control associated with DNA methylation states[14]. The majority of human epigenetic studies of HIV-1 infection are limited in interpretation due to profiling whole blood and utilization of a cross-sectional study design. Whether DNA methylation changes associated with HIV-1 infection occur in all blood cell types or if they are cell-type specific remains understudied and a challenge for studies that lack cryopreserved biospecimens. Moreover, we are aware of no study which has examined cell-type specific DNA methylation profiles during the earliest stages of AHI and longitudinally following the immediate initiation of ART.

In this study, we examined whether alterations to cell-type specific DNA methylation occur early during AHI in monocytes and CD4^+^ T lymphocytes and whether the early initiation of ART restores epigenetic changes associated with AHI. We identified the earliest host epigenetic changes to genome-wide DNA methylation from purified cell-sorted peripheral monocytes and CD4^+^ T lymphocytes from participants in the RV254/SEARCH 010 acute HIV-1 cohort that enrolls acutely infected individuals who initiate ART within 3 days after diagnosis. Moreover, we determined that the early initiation of ART minimally restores acute HIV-related DNA methylation changes in monocytes at interferon-related genes and had no impact on DNA methylation changes associated with AHI in CD4^+^ T lymphocytes. We also explored whether AHI-associated DNA methylation changes in monocytes and CD4^+^ T lymphocytes at the earliest stages of acute HIV-1 infection were associated with clinically relevant outcomes following ART. Defining the earliest impact of HIV-1 on the host epigenome and identifying indelible epigenetic marks embedded by HIV-1 infection and ART is key to understanding disease pathology and associated comorbidities, and to overcome barriers to cure related to HIV-1 persistence.

## Results

### Acute HIV-1 infection associates with early DNA methylation changes in monocytes and CD4^+^ T lymphocytes

To define cell-type specific genome-wide DNA methylation changes that relate to AHI, we first compared the methylomes of fluorescence-activated cell sorted monocytes and CD4^+^ T lymphocytes from 22 male Thai individuals living with acute HIV-1 infection in the RV254/SEARCH010 cohort (Fiebig stages I to V; median days since estimated exposure was 17.5 days) who were ART naive and 8 demographically matched HIV-1 uninfected Thai participants. Four participants were in the Fiebig stage I of infection corresponding to detectable HIV-1 nucleic acid and the absence of p24 antigen. The earliest captured data profiled related to a participant in Fiebig stage I with estimated 9 days since exposure. All individuals with AHI were infected with HIV-1 CRF01-AE. Detailed study participant characteristics are presented in **Table 1**.

**Table 1:** Clinical characteristics of study participants upon diagnosis and enrollment.

	HIV- (n = 8)		AHI (n = 22)	
		Fiebig I	Fiebig II	Fiebig III-V
Age (year)^a^	30.5 (25–39)	26 (22–42)	22 (19–44)	27 (21–42)
Sex (male, %)	100	100	100	100
Enroll HIV-1 Viral Load (Log_10_) ^a^	-	3.94 (3.65–4.06)	5.82 (4.83–6.96)	5.55 (4.9–7.43)
Days of Infection (days) ^a^	-	14 (9–19)	16 (14–19)	18 (8–28)
Enroll CD4 T (cells/uL) ^a^	-	603 (354–761)	342 (165–773)	476 (181–736)
CD8 T (cells/uL) ^a^	-	424 (189–879)	308 (201–1644)	1008 (496–2434)
CD4/CD8 Ratio^a^	-	1.67 (0.64–1.87)	0.74 (0.27–1.70)	0.47 (0.17–1.16)
Subtype CRF_01AE (%)	-	4 (100%)	5 (100%)	13 (100%)

^a^Data are median (interquartile range, IQR)

**Fig 1:**
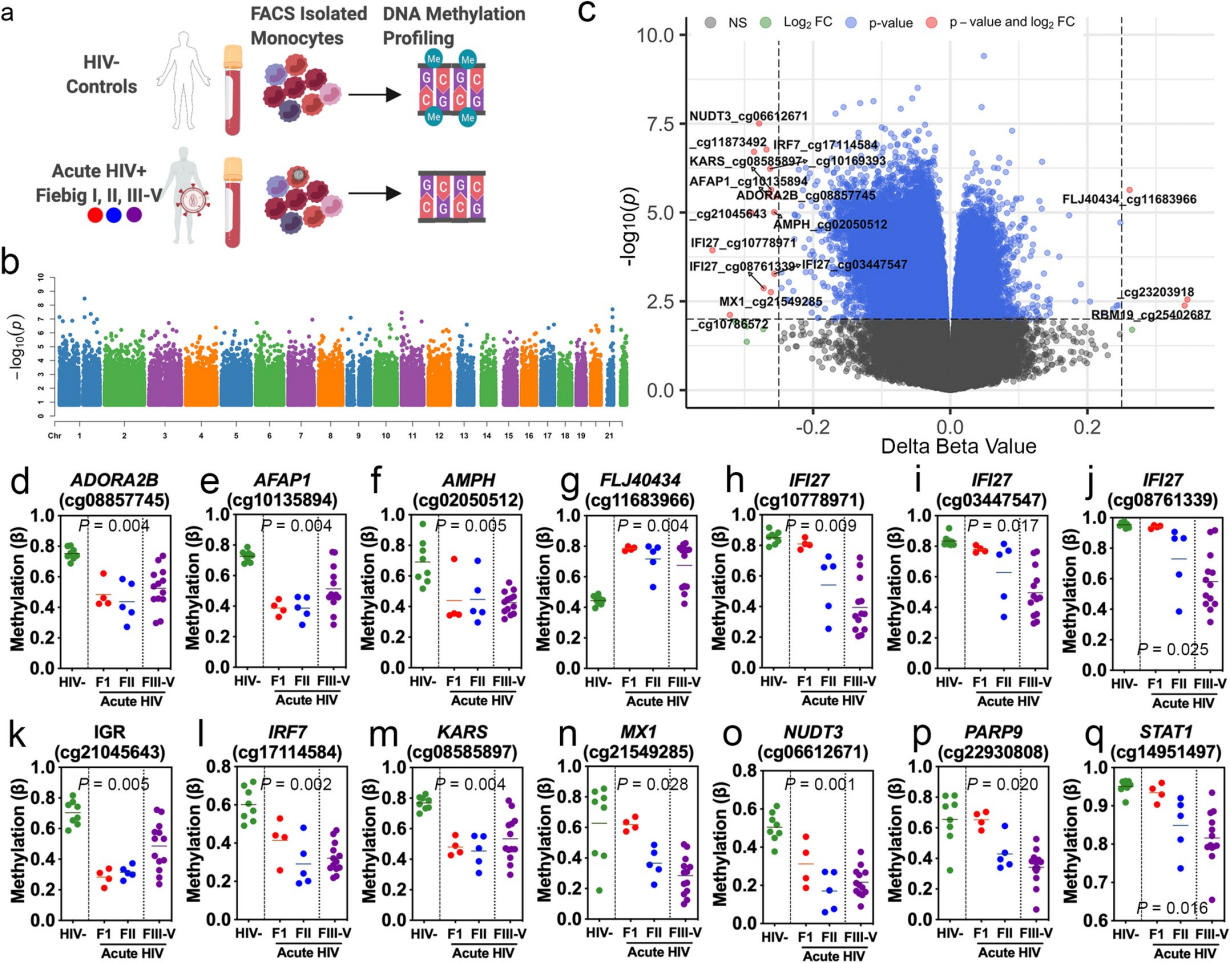
Monocyte cell type-specific differentially methylated loci associated with acute HIV infection. (a) Diagram of experimental design. Created with BioRender.com (b) Manhattan plot of differentially methylated loci associated with AHI in monocytes displayed across chromosomes. P values transformed using -log10(P) (c) Volcano plot displaying HUGO gene symbols and related CpG ID (cg#) for top hypomethylated and hypermethylated sites. Difference in DNA methylation displayed as delta beta values plotted against P values transformed using -log10(P). NS: non-significant. (d-q) Plots demonstrating changes in DNA methylation in AHI Fiebig I (red), Fiebig II (blue) Fiebig III-V (purple) compared to HIV- (green) at single CpGs sites related to specific genes or intergenic regions of the genome displayed in bold italic above CpG ID (cg#). Significance determined comparing HIV- vs. AHI and using FDR adjusted P-values <0.05.

**Fig 2:**
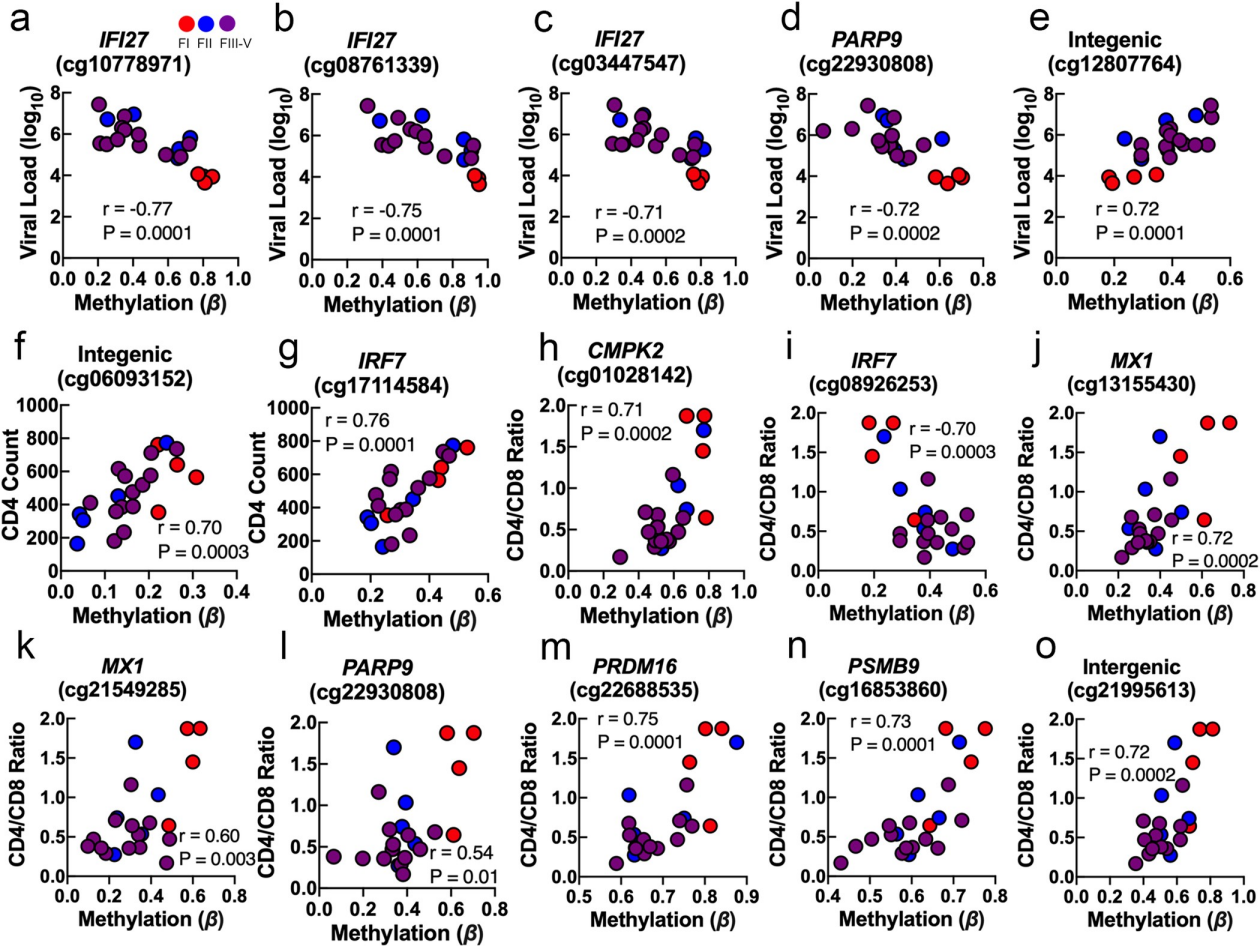
Associations of monocyte cell type-specific DNA methylation with viral load, CD4 count, and CD4/CD8 ratio. Scatter plots showing the correlation between (a-e) plasma viral load, (f-g) pre-ART CD4 count, and (h-o) pre-ART CD4/CD8 ratio and DNA methylation levels at single CpGs sites related to specific annotated protein-coding genes displayed in bold italic above CpG ID (cg#) in monocyte cells during AHI at entry. P-values were calculated with the Spearman correlation test.

In our first step, we examined whether the greatest number of differentially methylated loci (DML) related to AHI were present in total monocytes or CD4^+^ T lymphocytes by using a cross-sectional analysis of genome-wide DNA methylation data obtained from AHI and HIV-1 uninfected participants. We observed the greatest number of DML related to AHI (22,697 loci) in total monocytes compared to CD4^+^ T lymphocytes (294 loci) at an absolute mean difference in methylation greater than 5% between AHI and HIV-1 uninfected (Δβ-value > |0.05| and significant at FDR adjusted *P* < 0.05) (**Fig 1A and S1 Data**). In monocytes, the distribution of DML across the human genome was not biased to a specific chromosome, suggesting broad impacts of AHI upon the DNA methylation landscape of monocytes (**Fig 1B**). Notably, the majority of monocyte-specific DML related to AHI were hypomethylated in AHI participants compared to uninfected participants (96.41%; 21,883 CpGs), suggestive of a transcriptionally active epigenetic state related to innate immune activation during viral infection (**Fig 1C**). We examined whether these DML related to AHI in monocytes were enriched in specific genomic regions linked to gene regulation and found a significant enrichment in intergenic (odds ratio [OR] = 1.3; *P* = 0.0000007), gene body (OR = 1.2; *P* = 0.0003), and open sea CpG island regions (OR = 1.2; *P* = 0.0003) compared to the expected distribution of methylation sites assayed across the human genome (**S1 Table**). Moreover, when we examined the overlap of the top 1000 DML associated with AHI in monocytes with a 15 state chromatin state model derived from five histone modification marks for primary monocytes generated from the NIH Roadmap Epigenomics Consortium[15], we observed a significant enrichment in a transcribed chromatin state at the 5’ and 3’ end of genes showing both promoter and enhancer signatures (TxFlnk; OR = 4.6; *P* = 0.005), suggesting the DML associated with AHI in monocytes occur at distinct regulatory regions (**S2 Table**). Gene annotation and enrichment analyses of the top 1000 DML associated with AHI in monocytes revealed the top gene ontology enrichment in defense response to virus (*P* = 0.008) (**S3 Table**). This finding supports the important early role of innate immune activation in AHI and suggest demethylation of regulatory regions of specific genes is critical to innate immune activation during AHI. The top Δβ-value methylation changes were observed at loci related to the following genes: *ADORA2B* (cg08857745), *AFAP1* (cg10135894), *AMPH* cg02050512, *FLJ40434* (cg11683966), *IFI27* (cg10778971, cg03447547, cg08761339), intergenic region (cg21045643), *IRF7* (cg17114584), *KARS* (cg08585897), *MX1* (cg21549285), *NUDT3* (cg06612671), *PARP9* (cg22930808), and *STAT1* (cg14951497) (**Fig 1D–1Q**). We observed that DNA methylation levels at loci related to interferon genes including *IFI27* (**Fig 1H–1J**), *IRF7* (**Fig 1l**), *MX1* (**Fig 1N**), *PARP9* (**Fig 1P**), and *STAT1* (**Fig 1Q**) appeared to vary depending on Fiebig stage. Specifically, levels of DNA methylation related to interferon genes in AHI participants identified as Fiebig stage I appeared more similar to uninfected controls than the DNA methylation levels for AHI participants Fiebig stage II-V, suggesting a link between HIV-1 viral sensing by monocytes and interferon gene demethylation. Therefore, we decided to examine whether the level of DNA methylation at interferon-related genes significantly correlated with participants plasma viral load. Indeed, participant’s viral load at study entry was associated with monocyte DNA methylation levels at five DML including two interferon-related genes (*IFI27*, cg10778971: r = -0.77, *P* = 0.0001; *IFI27*, cg08761339: r = -0.75, *P* = 0.0001; *IFI27*, cg03447547: r = -0.71, *P* = 0.0002; *PARP9*, cg22930808: r = -0.72, *P* = 0.0002; and intergenic region, cg12807764: r = 0.72, *P* = 0.0001) (**Fig 2A–2E**).

Next, we sought to further explore whether monocyte DNA methylation patterns associated with AHI related to participants’ immune system health determined by CD4 count and CD4/CD8 ratio. We observed that participants’ CD4 count at study entry was significantly associated with two monocyte DML at an intergenic region (cg06093152: r = 0.70, *P* = 0.0003) and the interferon regulatory factor 7 (*IRF7*) gene that is involved in transcriptional activation of virus-inducible host genes (cg17114584: r = 0.76, *P* = 0.0001) controlling for viral load (**Fig 2F–2G**). Participants’ CD4/CD8 ratio at study entry was associated with eight DML (*CMPK2*, cg01028142: r = 0.71, *P* = 0.0002; *IRF7*, cg08926253: r = -0.70, *P* = 0.0003; *MX1*, cg13155430: r = 0.72, *P* = 0.0002; *MX1*, cg21549285: r = 0.60, *P* = 0.003; *PARP9*, cg22930808: r = 0.54, *P* = 0.01; *PRDM16*, cg22688535: r = 0.75, *P* = 0.0001; *PSMB9*, cg16853860: r = 0.73, *P* = 0.0001; intergenic region (cg21995613): r = 0.72, *P* = 0.0002) (**Fig 2H–2O**). We found no statistically significant associations between monocyte DNA methylation patterns related to AHI and estimated duration since exposure obtained from questionnaire and medical history.

**Fig 3:**
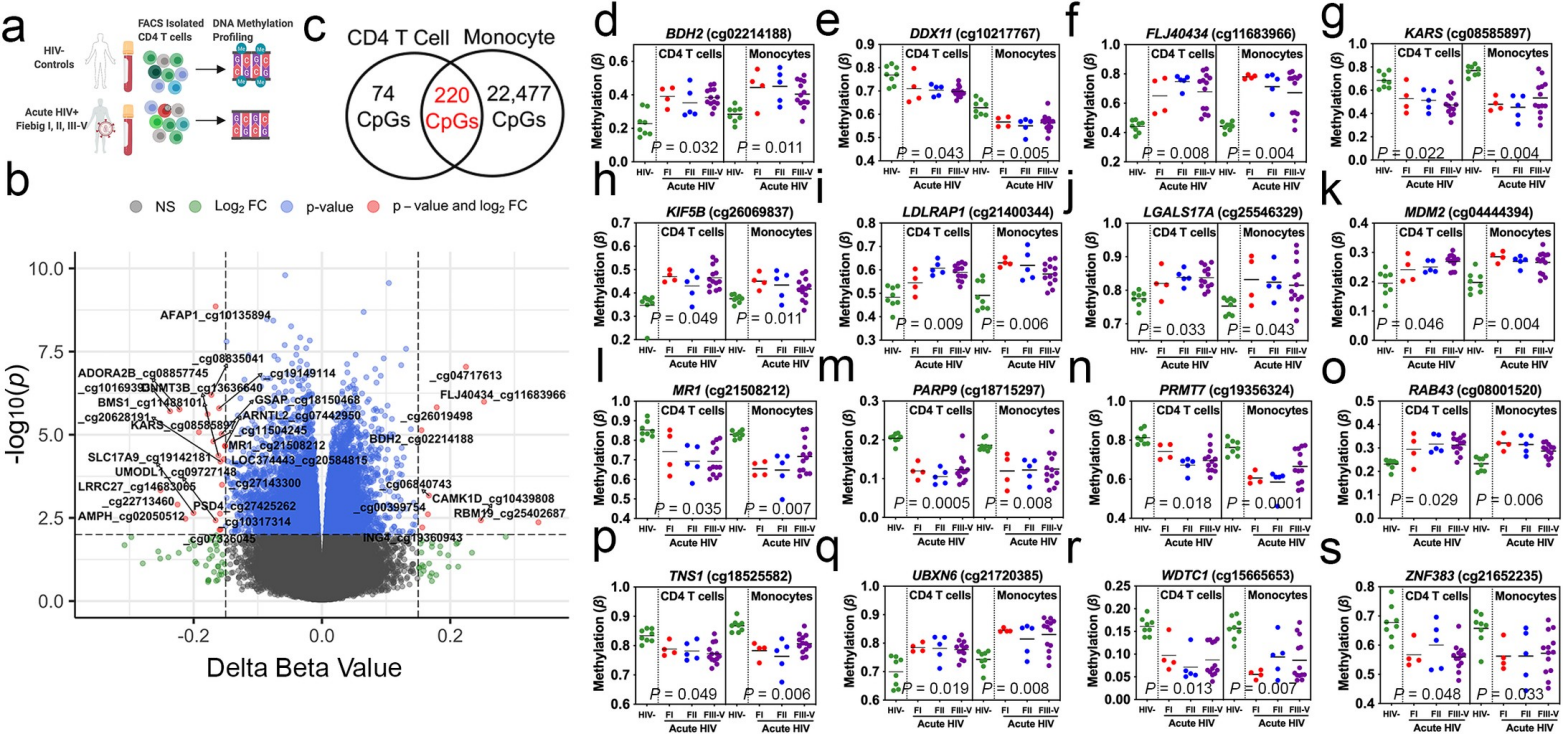
Cell-type independent differentially methylated loci associated with acute HIV infection. (a) Diagram of experimental design displaying comparison utilized to identify DML in CD4+ T cells associated with AHI. Created with BioRender.com (b) Volcano plot displaying gene symbol and CpG ID (cg#) for top hypomethylated and hypermethylated sites associated with AHI in CD4^+^ T lymphocytes. Difference in DNA methylation displayed as delta beta values plotted against P values transformed using -log10(P). (c) Venn diagram displaying the number of overlapping DML and cell-type specific DML identified in CD4+ T cells and monocyte cells (d-s) Plots demonstrating changes in DNA methylation in AHI Fiebig I (red dots), Fiebig II (blue dots) Fiebig III-V (purple dots) participants compared to HIV- (green dots) at single CpGs sites related to annotated protein-coding genes displayed in bold italic above CpG ID (cg#) for CD4^+^ T lymphocytes and monocytes. Significance determined comparing HIV- vs. AHI and using FDR adjusted P-values <0.05.

Examining genome-wide DNA methylation of sorted CD4^+^ T lymphocytes from the same participants (**Fig 3A**), we observed 294 differentially methylated loci (DML) associated with AHI in CD4^+^ T lymphocytes at an absolute mean difference in methylation greater than 5% between AHI and HIV uninfected (Δβ-value > |0.05| and significant at FDR adjusted *P* < 0.05) (**Fig 3B** and **S2 Data**). Similar to monocytes, the majority DML that differed by 5% were hypomethylated in HIV-infected compared to uninfected monocytes (66.66%; 196 CpGs), suggestive of a transcriptionally active epigenetic state associated with AHI (**S1 Fig**). We examined whether the DML related to AHI in CD4^+^ T lymphocytes were enriched in specific genomic regions and found a significant enrichment in CpG island (OR = 1.5; *P* = 0.005) and transcription start site (TSS) to– 200 nucleotides upstream of the TSS (TSS200) (OR = 1.4; *P* = 0.04) compared to the expected distribution of methylation sites assayed across the human genome (**S4 Table**). Additionally, when we examined the overlap of the top 294 DML associated with AHI with a 15 state chromatin state model derived from five histone modification marks for primary T cell populations generated from the NIH Roadmap Epigenomics Consortium[15], we observed an enrichment in a chromatin state associated with zinc finger protein genes (ZNF/Rpts; OR = 7.05; *P* = 0.009), transcribed states (Tx; OR = 2.6; *P* = 0.001), and proximal promoter states (TssA; OR = 1.9; *P* = 0.0001) (**S5 Table**). While the enrichment of CD4^+^ T lymphocytes DML at specific genomic regions contrasted with monocytes, we observed that 220 DML associated with AHI identified in CD4^+^ T lymphocytes overlapped with DML we had identified in monocytes (**Fig 3C and S3 Data**). This finding suggest that a subset of DNA methylation changes associated with AHI in blood may be cell-type independent. Examples of overlapping AHI-related DML in CD4^+^ T lymphocytes and monocytes included sites related to the following genes *BDH2* (cg02214188), *DDX11* (cg10217767), *FLJ40434* (cg11683966), *KARS* (cg08585897), *KIF5B* (cg26069837), *LDLRAP1* (cg21400344), *LGALS17A* (cg25546329), *MDM2* (cg04444394), *MR1* (cg21508212), *PARP9* (cg18715297), *PRMT7* (cg19356324), *RAB43* (cg08001520), *TNS1* (cg18525582), *UBXN6* (cg21720385), *WDTC1* (cg15665653), and *ZNF383* (cg21652235) (**Fig 3D–3S**). We observed for a subset of DML that participants at the earliest stages of infection in Fiebig stage I displayed alterations to methylation at these DML similar to later Fiebig stages in both CD4^+^ T lymphocytes and monocytes, suggesting these AHI-related DNA methylation changes occur rapidly and prior to a robust host immune response. These data support findings in RV254/SEARCH010 showing that HIV-1 rapidly establishes during the earliest stage of AHI (Fiebig I)[16,17]. Moreover, the directionality of change for the cell-type independent DML associated with AHI (hypo- or hyper-methylation) was similar in CD4^+^ T lymphocytes and monocytes (**Fig 3D–3S**). Unsupervised hierarchical clustering based on these 220 AHI-related DNA methylation differences distinguished the majority of participants living with AHI from uninfected controls (**S2 Fig**), further supporting the suggestion that cell-type independent AHI-related changes to a subset of DNA methylation loci occur early and for both innate and adaptive immune cell types.

### Early initiation of ART during AHI minimally restores DNA methylation changes in monocytes linked to interferon-related genes and has no impact in CD4^+^ T lymphocytes

**Fig 4:**
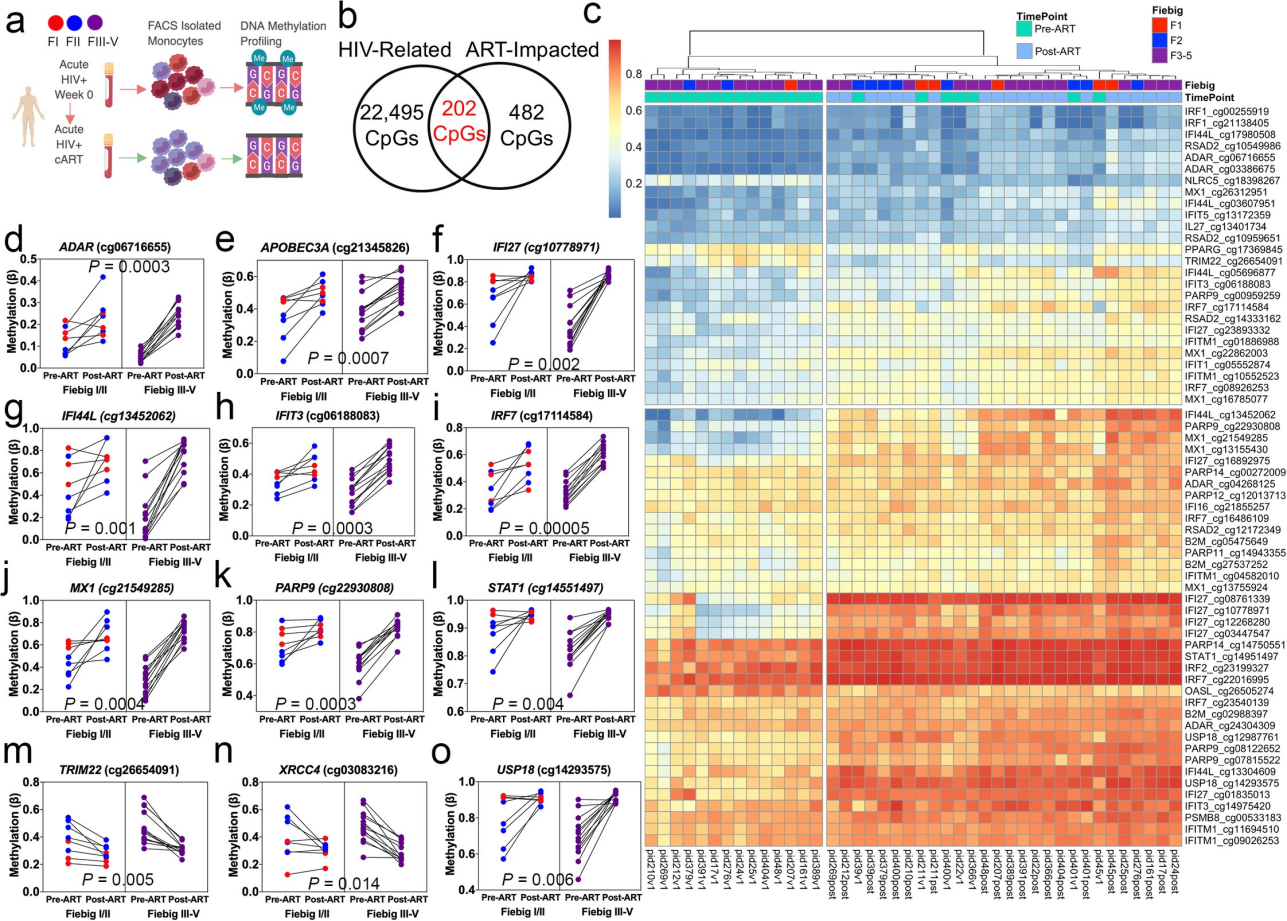
Early initiation of ART during acute infection minimally impacts monocyte cell type-specific differentially methylated loci associated with acute HIV infection. (a) Diagram of longitudinal experimental design utilized to identify DML-related to ART treatment in purified monocytes. Created with BioRender.com (b) Venn diagram displaying the overlap of DML between DNA methylation sites identified in monocytes related to AHI and DNA methylation sites identified in monocytes that changed before and after early initiation of ART during AHI. (c) Heatmap showing the unsupervised clustering of DML at annotated protein-coding genes related to interferon at pre-ART (aqua color) and post-ART (light blue) timepoints for AHI participants staged at Fiebig I (red color), II (blue color), or III-V (purple color). Dendrogram shown above. Colors in the heatmap indicate CpG methylation levels (blue to red: low to high methylation levels). (d-o) Plots displaying longitudinal DNA methylation levels of AHI participants at Pre-ART and Post-ART timepoints by Fiebig stage. Red dots represent individuals in Fiebig I. Significance determined comparing repeated measures comparison of Pre-ART vs. Post-ART and using FDR adjusted P-values <0.05.

A unique feature of the RV254/SEARCH010 AHI cohort is the early administration of ART within 3 days of participant enrollment into the cohort and the multiple biospecimen collections performed[18], permitting longitudinal study analyses. Thus, we evaluated whether DNA methylation profiles in monocytes and CD4^+^ T lymphocytes of 21 AHI participants profiled prior to ART initiation were impacted by early initiation of ART by comparing baseline pre-ART and post-ART timepoints (**Fig 4A**). In this longitudinal assessment of DNA methylation, the median days of infection was 16 days for the baseline pre-ART timepoint examined and 232 days for the post-ART timepoint. All participants were virally suppressed and were undetectable (plasma HIV-1 Viral Load <50 copies/ml) at the timepoint assayed for DNA methylation post-ART. We utilized a paired differential methylation analysis and observed 684 DML for monocytes and 0 DML for CD4^+^ T lymphocytes showing greater than 5% absolute mean differences between pre- and post-ART time points (Δβ-value > |0.05| and significant at FDR adjusted *P* < 0.05 (**S4 Data**). Less than 1% of DML (202 sites) in monocytes that we had associated with AHI overlapped with DML impacted by the early initiation of ART (**Fig 4B**), suggesting ART minimally restored DNA methylation changes to a state present in uninfected individuals.

Our data suggested that early initiation of ART during AHI appeared to have no impact on CD4^+^ T lymphocytes DNA methylation states within a year of treatment as we did not observe any significant differences in DNA methylation at the site level comparing pre-ART and post-ART for the 21 AHI participants. We sought to broaden our analysis of the epigenetic dataset for CD4+ T lymphocytes based on previous work that has shown DNA methylation levels at a subset of genomic loci accurately predict age termed the “epigenetic clock”[19] and chronic HIV-1 infection accelerates epigenetic age by approximately 5 years despite sustained therapy[13,20]. We calculated epigenetic age acceleration estimates for matched CD4+ T lymphocytes and monocytes from participants at pre-ART and post-ART timepoints. During pre-ART AHI, we observed a significantly greater accelerated epigenetic age in CD4+ T lymphocytes compared to monocytes from the same participants (**S3 Fig**). ART significantly decreased epigenetic aging in the CD4 T cell compartment comparing pre-ART and post-ART epigenetic age acceleration estimates of participants (**S3 Fig**). In contrast, we did not observe any significant differences in age acceleration from monocytes comparing pre-ART and post-ART timepoints. The median age acceleration in monocytes increased from 3.6 to 5.8 years compared to 7.6 to 3.4 years for CD4+ T lymphocytes at pre-ART and post-ART timepoints. These data suggest that the epigenetic clock of CD4+ T lymphocytes is rapidly impacted and more vulnerable compared to monocytes during AHI. Furthermore, a benefit of early initiation of ART during AHI is lowering epigenetic age acceleration estimates in CD4+ T lymphocytes. Epigenetic age acceleration assessments in CD4+ T cells and monocytes of uninfected controls were significantly lower in comparative analyses to pre-ART and post-ART AHI participants (**S3 Fig)**. Our data add to recent findings in whole blood that after 2 years of ART initiation epigenetic ageing associated with untreated chronic HIV infection is partly reversed [21].

Focusing on the site-specific ART-related monocyte DNA methylation changes identified in our paired differential methylation analysis, we sought to examine whether ART-related DNA methylation changes were predominately increases or decreases in DNA methylation following ART. We observed an equal percentage of DML that became hypermethylated (54%; 371 CpGs) or hypomethylated (46%; 313 CpGs) following ART in monocytes, indicating that ART did not fully restore the majority hypomethylation phenotype we observed related to AHI in monocytes (**S4 Data**). We examined whether the DML were enriched in genomic regions we had identified for monocytes and found a significant enrichment in gene body (Odds Ratio = 1.4; *P* = 0.00005) and open sea CpG island regions (Odds Ratio = 2.2; *P* = 6.06E-21) compared to the expected distribution of methylation sites assayed across the human genome (**S6 Table**). The overlap of the 684 DML following ART with a 15 state chromatin state model derived from five histone modification marks for primary monocytes showed a significant enrichment in enhancer states (Enh; Odds Ratio = 10.4; *P* = 2.01E-176) and a transcribed state at the 5’ and 3’ end of genes showing both promoter and enhancer signatures (TxFlnk; Odds Ratio = 8.9; *P* = 2.5E-12) (**S7 Table**). We observed DNA methylation levels at loci related to interferon-related genes supported by gene ontology enrichment analyses (**S8 Table**) significantly increased comparing pre-ART and post-ART time points (*P* = 1.69E-07), supporting the transcriptional suppression of interferon responses in innate immune cells following viral suppression due to ART. 14 participants pre-ART could be stratified based on monocytes DNA methylation states related to interferon genes using unsupervised hierarchical clustering (**Fig 4C**). Examples of restoration of DNA methylation states associated with ART in monocytes that may vary based on Fiebig stage occurred at regulatory regions of *ADAR* (cg06716655), *APOBEC3A* (cg21345826), *IFI27* (cg10778971), *IFI44L* (cg13452062), *IFIT3* (cg06188083), *IRF7* (cg17114584), *MX1* (cg21549285), *PARP9* (cg22930808), *STAT1* (cg14551497), *TRIM22* (cg26654091, *XRCC4* (cg03083216), and *USP18* (cg14293575) (**Fig 4D–4O**). We observed a noticeable degree of variation among participants DNA methylation levels at both pre-ART and post-ART time points supporting the observation of individual differences in DNA methylation setpoints and dynamics at interferon genes in innate immune monocyte cells during AHI and post-ART (**S4 Data**).

**Fig 5:**
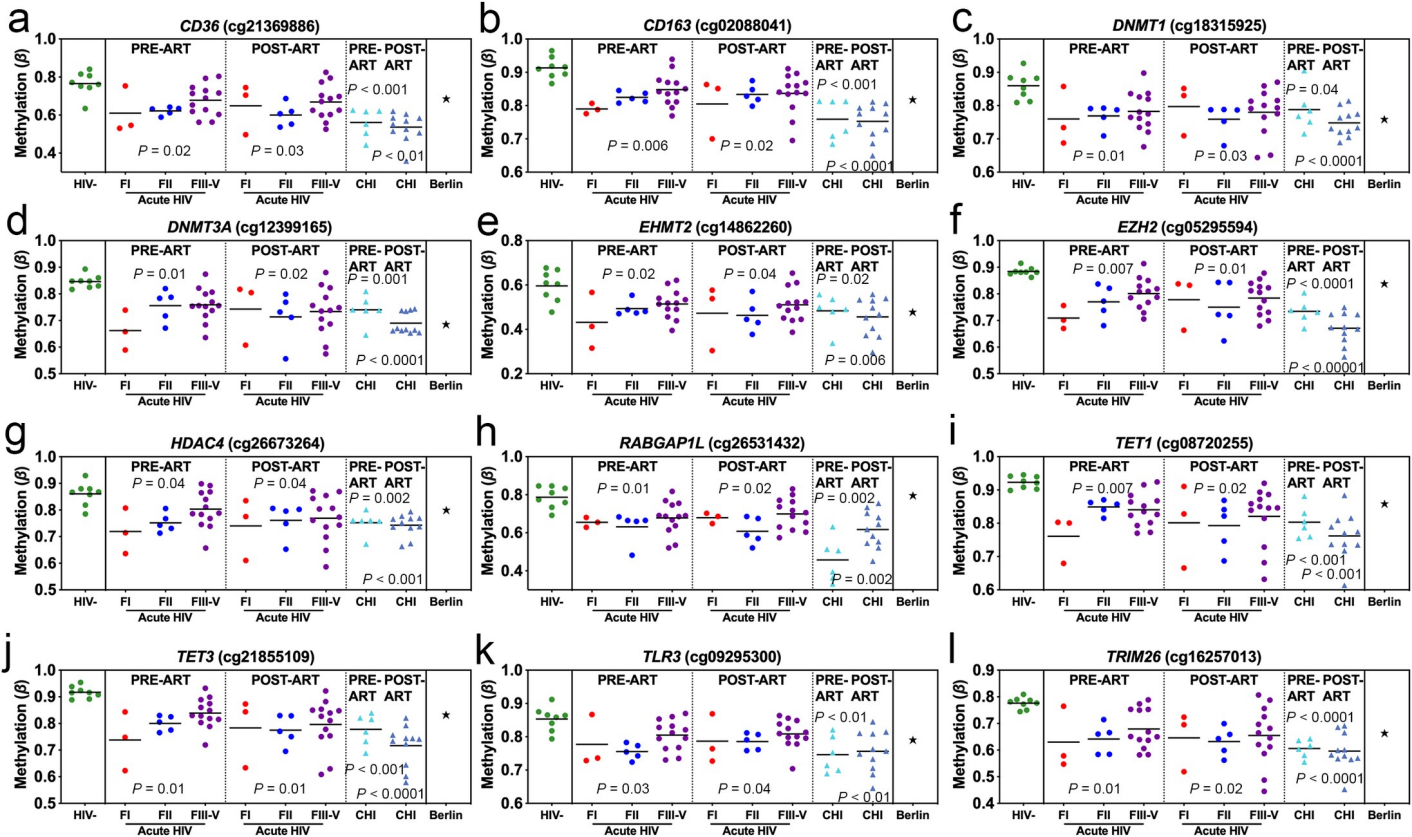
Differentially methylated loci identified in monocyte cells that persist in acute and chronic HIV infection despite ART. (a-l) Plots of durable differentially methylated loci in in AHI Fiebig I (red), Fiebig II (blue) Fiebig III-V (purple) at Pre-ART and Post-ART time points compared to HIV- (green) at single CpGs sites related to specific genes or intergenic regions of the genome displayed in bold italic above CpG ID (cg#). Validation of these same durable differentially methylated loci in monocytes displayed for chronic HIV infection at Pre-ART (aqua) and Post-ART (light blue) along with the Berlin patient (star). Significance determined comparing HIV- vs. AHI pre-ART, AHI post-ART, CHI pre-ART, and CHI post-ART and using FDR adjusted P-values <0.05.

Given that we had observed that the majority of DML associated with AHI in monocytes (22,495 DML, 99%) appeared to be durable methylation states defined by being unchanged comparing pre-ART and post-ART, we sought to confirm our findings and examined whether this was apparent in a longitudinal chronic HIV cohort. We chose 12 durable loci associated with AHI in monocytes and validated these loci in monocytes from a chronic HIV infection (CHI) Thai cohort that included pre-ART and post-ART biospecimens. Examples of durable methylation states associated with AHI and CHI in monocytes that were significantly different compared to uninfected controls but not significant comparing pre-ART and post-ART occurred at loci related to genes involved in innate immune cell antiviral and inflammation activity and DNA methylation deposition and erasure: *CD36* (cg21369886), *CD163* (cg02088041), *DNMT1* (cg18315925), *DNMT3A* (cg12399165), *EHMT2* (cg14862260), *EZH2* (cg05295594), *HDAC4* (cg26673264), *RABGAP1L* (cg26531432), *TET1* (cg08720255), *TET3* (cg21855109), *TLR3* (cg09295300), and *TRIM26* (cg16257013) (**Fig 5A–5L**). The methylation state of the Berlin Patient’s monocytes at these loci was more similar to uninfected participants compared to AHI and chronic HIV. The aberrant DNA methylation states associated with AHI in monocytes that are not impacted by the early initiation of ART in AHI nor during CHI may serve as a therapeutic target for myeloid epigenetic editing strategies.

### Monocyte transcription at interferon-related genes during AHI associate with DNA methylation states and are predominately downregulated following ART

**Fig 6:**
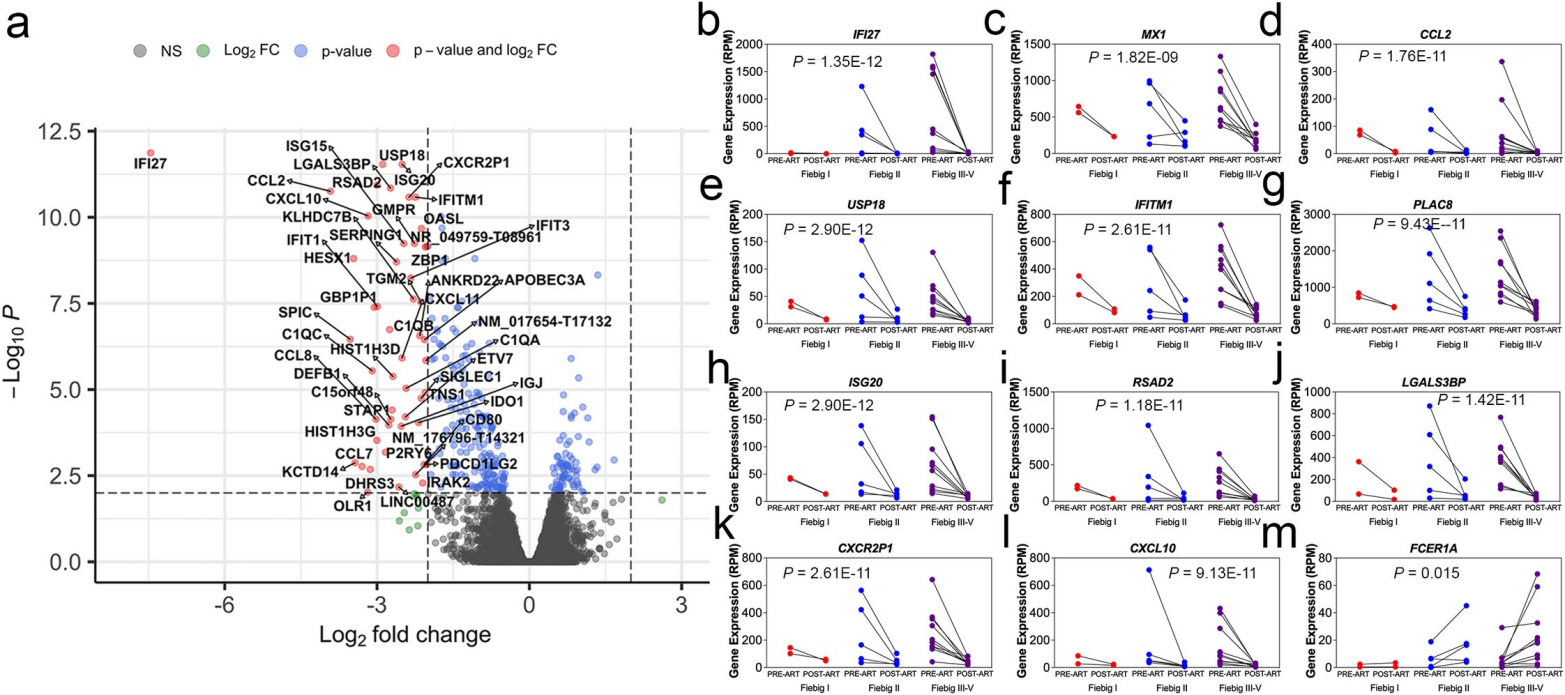
Early initiation of ART during acute infection impacts interferon-related gene programs in monocytes. (a) Volcano plot showing gene symbol for top differentially expressed genes in monocytes comparing pre-ART and post-ART time points. Log fold change plotted against transformed -log10 P value. (b-m) Plots showing differentially expressed interferon-related genes in monocyte cells by Fiebig stage comparing pre-ART and post-ART timepoints. Fiebig I displayed in red, Fiebig II displayed in blue, and Fiebig III-V displayed in purple.

**Fig 7:**
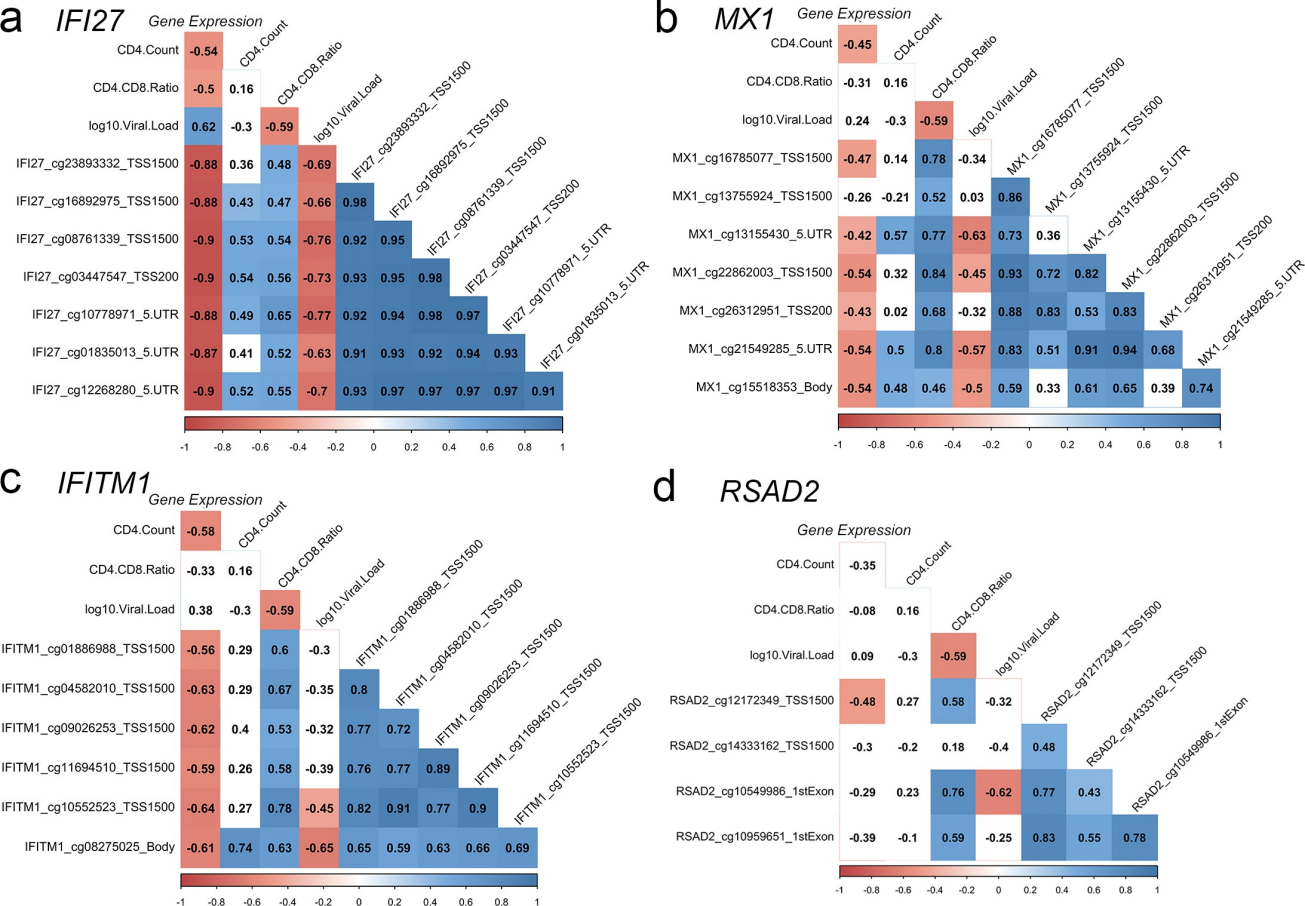
Monocyte gene expression associates with DNA methylation levels and clinical parameters during acute HIV infection. (a-d) Correlation matrix plots showing the correlation coefficient for associations between gene expression levels, CD4 count, CD4/CD8 ratio, viral load, and DNA methylation levels at specific genomic loci. Significant correlations displayed as solid colored boxes (red displayed for negative correlations and blue for positive correlations). Correlation coefficient displayed.

Based on an integrative analysis with chromatin state data for primary monocytes (CD14+; E029) from peripheral blood of a healthy donor from the Roadmap Epigenomics project[15], we observed that 2,361 of the DML we had identified associated with AHI in monocytes occurred in annotated enhancer, enhancer bivalent, or enhancer genic regions of the genome (**S1 Data**), suggesting the methylation differences occurred at regulatory regions of the genome for primary monocyte cells that would relate to transcriptional changes. Hence, we used targeted transcriptome profiling of 20,802 genes in monocytes from 17 AHI participants at pre-ART and post-ART timepoints matching those that we had assayed longitudinal DNA methylation. The differential expression analyses revealed 557 genes differentially expressed in monocytes between pre-ART and post-ART at an FDR <0.05 (**Fig 6A and S5 Data**). The majority (64%) of differentially expressed genes were downregulated following ART compared to only 36% upregulated (**Fig 6A**). Gene expression in monocytes decreased comparing pre-ART and post-ART for genes including *IFI27*, *MX1*, *CCL2*, *USP18*, *IFITM1*, *PLAC8*, *ISG20*, *RSAD2*, *LGAS3BP*, *IFITM1*, *CXCR2P1*, and *CXCL10* (**Fig 6B–6L**). Transcriptional upregulation of *FCER1A*, a gene involved in antigen presentation[22], was identified in monocytes comparing pre-ART and post-ART (**Fig 6M**). We observed that AHI participants in Fiebig stage I (red) showed levels of gene expression at interferon-related genes that did not drastically change comparing pre-ART and post-ART as in later Fiebig stages (blue and purple) supporting our observations for DNA methylation patterns (**Fig 6B–6L**). We identified that 79 genes that were differentially expressed in monocytes following ART overlapped with DNA methylation changes related to ART in AHI (**S6 Data**), suggesting these genes may be under epigenetic regulation involving DNA methylation. Correlation analyses showed that transcriptional levels of *IFI27* in monocytes during AHI pre-ART significantly associated with DNA methylation levels, CD4 count, CD4/CD8 ratio, and viral load (**Fig 7A**). Transcriptional levels of *MX1* in monocytes during AHI pre-ART significantly associated with DNA methylation levels and CD4 count (**Fig 7B**). Transcriptional levels of *IFITM1* in monocytes during AHI pre-ART significantly associated with DNA methylation levels and CD4 count (**Fig 7C**). Transcriptional levels of *RSAD2* significantly associated with DNA methylation levels at a genomic loci located within 1500 base pairs of the transcriptional start site (**Fig 7D**). Other notable relationships between transcriptional levels and DNA methylation levels occurred at genes including *APOBEC3A*, *AIM2*, *STAT1*, *XRCC4*, *PDE4B*, and *USP18* (**S4 Fig**).

### DNA methylation patterns in monocytes at the *IRF7* gene locus during AHI associates with longitudinal CD4 cell recovery following ART

**Fig 8:**
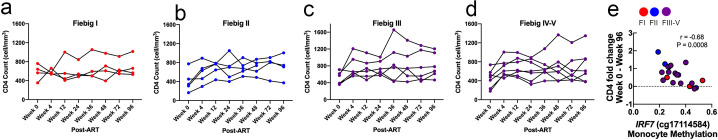
DNA methylation related to the *IRF7* gene in monocytes relates to CD4 fold change at 96 weeks following the initiation of ART during acute HIV infection. (a-d) Line plots of longitudinal CD4 count in participants by Fiebig stage from week 0 (pre-ART) through post-ART time points out to week 96. (e) Correlation plot displaying relationship between *IRF7* DNA methylation levels in monocyte cells assessed at baseline and CD4 fold change calculated from week 0 to week 96. Fiebig stage of participant displayed: Fiebig I displayed in red, Fiebig II displayed in blue, and Fiebig III-V displayed in purple.

A preexisting host epigenetic state and/or epigenetic changes induced during the earliest stages of AHI may embed a biological memory that could impact longitudinal clinical outcomes despite the early initiation of ART. We sought to test this hypothesis by examining whether the DML we had identified associated with AHI in monocytes related to CD4 cell recovery defined as the fold change following ART up to 96 weeks. As expected, we observed AHI participants increased CD4 T cell count comparing pre- (Median CD4 T cell count = 464 cells/uL) and post-ART (Median CD4 T cell count = 676 cells/uL) time points (*P* = 0.0001). Supporting our previous observations[23], a subset of participants in all Fiebig stages did not achieve optimal CD4 cell recovery after ART (**Fig 8A–8D**). We identified that a monocyte DNA methylation feature at the *IRF7* gene loci (cg17114584) significantly associated with CD4 T cell fold change from Week 0 to Week 96 (r = -0.68, *P* = 0.0008) (**Fig 8E**). This relationship was still significant after controlling for baseline CD4 T cell count (r = -0.45, *P* = 0.025). Notably, we did not observe a significant relationship between methylation levels of cg17114584 and CD4 T cell fold change early at Week 12 or at Week 96 when looking at DNA methylation levels in participants CD4+ T lymphocytes (**S5 Fig**). *IRF7* encodes for a key innate immune molecule in the type I IFN signaling pathway and has been shown to control HIV-1 replication in macrophages[24]. These findings support findings showing the dynamic interplay between the innate immune system and CD4 T cell reconstitution and recent work highlighting monocyte features associating with poor CD4 T cell recovery after suppressive ART in chronically HIV-1 infected individuals [25].

### DNA methylation patterns in monocytes during acute HIV may relate to unfavorable clinical phenotypes and neurocognitive trajectories 96 weeks after treatment

**Fig 9:**
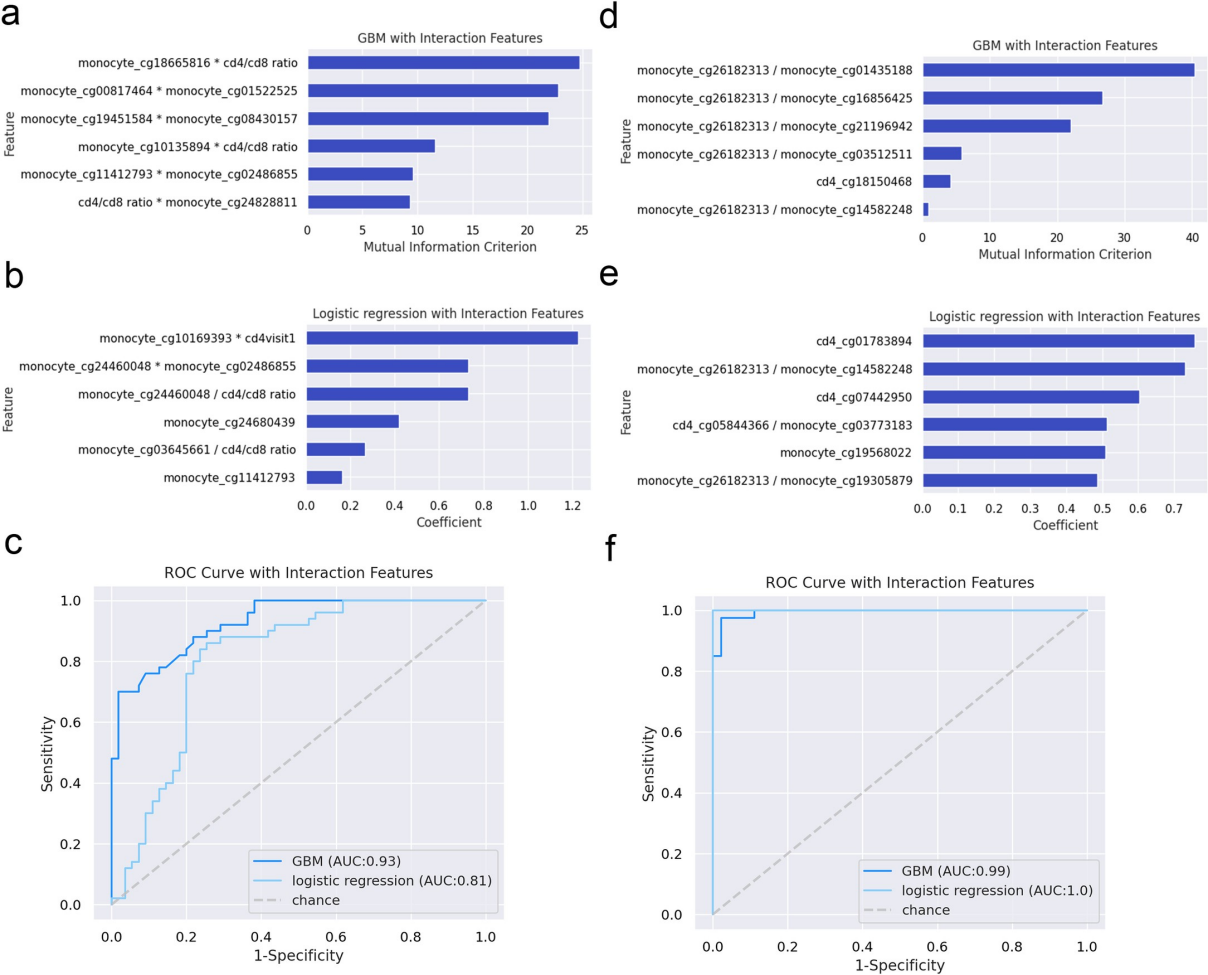
DNA methylation features associated with AHI in monocytes and CD4^+^ T lymphocytes predict favorable clinical phenotypes and neurocognitive performance following ART. (a-b) Feature importance plots of gradient boosting machine learning and logistic regression models with interaction features for predicting favorable clinical phenotype at week 96 post-ART utilizing cell-type specific DNA methylation measures and clinical parameters (estimated days of infection, viral load, CD4 count, CD8 count, and CD4/CD8 ratio) during viral establishment in AHI prior to ART initiation. (c) Receiver operating characteristic curve showing performance of classification models (area under curve AUC) in discovery AHI DNA methylation feature set. (e-f) Feature importance plots of gradient boosting machine learning and logistic regression models with interaction features for predicting neurocognitive performance trajectory at week 96 post-ART. (g) Receiver operating characteristic curve showing performance of classification models.

The RV254/SEARCH010 AHI cohort obtains extensive longitudinal clinical outcome measures including neurocognitive assessments [26–28]. Hence, as a discovery cohort analysis we sought to examine whether our AHI-associated epigenetic features were predictive of unfavorable clinical phenotypes and neurocognitive trajectories after ART. We categorized participants into those that showed an unfavorable clinical phenotype or achieved a favorable clinical phenotype after 96 weeks on ART based on fulfilling the following criteria for a favorable phenotype: no serious clinical events, VL <20 copies/ml at every visit after week 24, latest CD4 T cell count greater than 500 cells/mm^3^, and latest CD4/CD8 T cell ratio greater than 1 [29–33]. We identified that 11 AHI participants achieved a favorable clinical phenotype and 10 were unfavorable at week 96. We combined the 22,771 DML associated with AHI from monocytes and CD4^+^ T lymphocytes with clinical features (pre-ART CD4, CD8 T cell count, CD4/CD8 T cell ratio, viral load, and estimated days of infection) and utilized both a logistic regression with interaction features and gradient boosting machine learning method with a five-fold cross validation with five repeated trials (a total of 25 validation trials) to identify pre-ART AHI-related and/or host epigenetic features that predicted a favorable clinical phenotype at week 96. We identified that specific monocyte DNA methylation features (cg18665816, cg00817464, cg01522525, cg19451584, cg08430157, cg10125894, cg11412793, cg02486855, cg24828811, cg10169393, cg24460048, 02486855, cg24460048, cg24680439, cg03645661, and cg11412793) interacting with well-known clinical features of CD4 T cell count and CD4/CD8 T cell ratio predicting a AHI Thai participant having a favorable clinical phenotype at week 96 post-ART in both models (**Fig 9A and 9B**). The performance metrics of both gradient boosting machine learning and logistic regression with interaction features were comparable with area under the receiver operating characteristic curve above 0.8 in the validation dataset (**Fig 9C**). Of note, CD4^+^ T lymphocytes DNA methylation features, estimated duration since exposure, baseline CD8 count, and baseline viral load were not identified as significant features to predict unfavorable outcomes at week 96 following ART in AHI. This discovery epigenetic host feature set of predictive favorable clinical phenotypes following ART warrants investigation at longer post-ART time intervals and in other AHI cohorts with extensive longitudinal clinical outcome data collection.

Our previous research in the RV254 cohort showed that cognitive performance improves after treatment in AHI and the trajectory of improvement varies between participants[26]. Hence, we sought to examine whether AHI participants cognitive trajectories after ART could be tracked based on baseline pre-ART AHI-associated epigenetic features derived from specific immune cell types. Seventeen participants that had matching monocytes and CD4^+^ T lymphocytes DNA methylation datasets had undergone a comprehensive neuropsychological test battery longitudinally permitting the assessment of neuropsychological tests performance between baseline and 96 weeks following ART (see Materials and Methods). We dichotomized 9 participants into a high performing neurocognition group and 8 participants in a low performing neurocognition group based on the change in global neuropsychological tests z-scores (NPZ global) over 96 weeks on ART being greater than or less than the median NPZ global for the group. Using our cell-type epigenetic DNA methylation and clinical data feature set and a logistic regression with interaction features and gradient boosting machine learning method with a five-fold cross validation with five repeated trials (a total of 25 validation trials), we examined which pre-ART AHI features tracked with a low or high performing neurocognitive trajectory at 96 weeks post-ART. Both AHI-associated DML in monocytes (cg26182313, cg01435188, cg16856425, cg21196942, cg03512511, cg14582248, cg05844366, cg03773183, cg19568022, cg19305879) and CD4^+^ T lymphocytes (cg18150468, cg01783894, and cg05844366, and cg07442950) were identified as significant features tracking participant’s neuropsychological performance following ART in both models (**Fig 9D and 9E**). Clinical features including baseline CD4 T cell count, baseline CD8 T cell count, baseline CD4/CD8 T cell ratio, estimated duration since exposure, and baseline viral load were not identified as significant features to predict neuropsychological performance at week 96 following ART in AHI. Performance metrics of both gradient boosting machine learning and logistic regression with interaction features were comparable with area under the receiver operating characteristic curve above 0.9 in the validation trial dataset (**Fig 9F**). This discovery analysis suggest the utility of assessing host DNA methylation states to monitor brain health in HIV infection and support recent work highlighting the utility of DNA methylation in relationship to structural brain alterations observed in adolescents perinatally infected with HIV[34].

## Discussion

In this study, we examined the earliest *in vivo* effects associated with AHI on the epigenetic mechanism DNA methylation in purified cell-sorted peripheral monocytes and CD4^+^ T lymphocytes. Our findings reveal that significant differences in DNA methylation associated with AHI occur early in peripheral monocytes and CD4^+^ T lymphocytes. Notably, we observed that monocytes contain a markedly higher frequency of DML associated with AHI compared to CD4^+^ T lymphocytes. We observed that DNA methylation patterns at interferon-related genes in monocyte cells associate with plasma viremia, CD4 T cell count, and Fiebig stage of infection, highlighting the utility of patient specific epigenetic signatures as biomarkers. Additionally, we examined whether early treatment with ART initiated during AHI was associated with restorative changes in methylation profiles. Despite early treatment in these individuals, most DML observed during AHI were not restored to levels observed in uninfected controls. Of note, we found that early treatment was beneficial for reducing an epigenetic biomarker of accelerated aging in CD4+ T lymphocytes. Our data provide important evidence that the early host epigenetic changes related to AHI in HIV-1-relevant purified immune cell populations are minimally impacted by the early initiation of ART. Lastly, we identify that specific methylation states of monocytes during AHI link to important clinical states as measured by viral load burden, CD4 count, CD4/CD8 T cell ratio, longitudinal CD4 T cell recovery, favorable clinical phenotypes after ART, and neurocognitive performance trajectories after ART. Together, this discovery epigenetic dataset of AHI has important implications for HIV viral persistence, HIV cure strategies, and suggest determining epigenetic signatures prior to ART to guide precision HIV medicine.

The RV254/SEARCH 010 AHI cohort has revealed that ART initiated during acute HIV-1 infection rapidly decreases viral burden[35], limits virologic failure[36], and reduces viral reservoir size [16]. Despite the benefits of initiating ART at the earliest time possible following HIV-1 infection, other study findings from RV254/SEARCH 010 show that suboptimal CD4 T cell recovery still occurs in a small subset of individuals[23], early ART treatment during AHI only partially mitigates systemic immune inflammation[37], and rapid HIV RNA rebound occurs after ART interruption in individuals durably suppressed at the earliest stages of AHI[17]. Our findings add to previous work by suggesting that DNA methylation states in both monocytes and CD4^+^ T lymphocytes are rapidly impacted during AHI and initiating ART at the earliest time possible following HIV-1 infection does not mitigate the majority of changes to the host immune cellular epigenome. Whether HIV-1 targets monocytes and CD4^+^ T lymphocytes DNA methylation for viral persistence to evade immune detection and clearance during analytic treatment interruption in AHI remains an unknown question warranting investigation.

Our study findings support the hypothesis that HIV-1 infection rapidly compromises the epigenetic machinery of immune cells^26^. These findings also support a growing body of evidence demonstrating that the host epigenome is drastically impacted by HIV-1 infection and is associated with disease progression[14,38]. Previous studies utilizing comparative genome-wide DNA methylation profiling of blood from chronically infected individuals living with HIV have identify distinct DNA methylation signatures associated with chronic HIV[7] and HIV-related comorbidities and coinfections [8–11]. Longitudinal human DNA methylation studies of HIV in specific cell-types are rare and no study has examined DNA methylation states of individuals at the earliest stages AHI. Our study contributes to the epigenetic HIV-1 literature and advances previous DNA methylation profiling studies of HIV-1 by contributing the following: 1. A genome-wide DNA methylation dataset of AHI with participants identified at the earliest point in infection (Fiebig I); 2. A cell type specific genome-wide DNA methylation dataset of AHI from cell sorted monocytes and CD4^+^ T lymphocytes; and 3. Longitudinal data of AHI following the initiation of ART with clinical outcome measures.

The most distinct DNA methylation signal associated with acute HIV infection in monocytes appeared related to the interferon alpha inducible protein 27 (*IFI27*) gene. *IFI27* codes for a protein that is involved in type-I-interferon-induced apoptosis and antiviral activity. Previous research shows that *IFI27* gene expression is elevated in monocytes from HIV-suppressed HCV coinfected compared to HCV mono-infected participants[39] and *in vitro* infection of monocyte derived macrophages with HIV-1 downregulates *IFI27* gene expression[40]. Our findings demonstrate that the methylation state of the *IFI27* gene loci taken from monocyte cells during AHI was able to inform 4 key features. First, the Fiebig stage of a participant could be inferred by examining whether demethylation had occurred at this loci, with Fiebig I methylation appearing more similar to uninfected controls than later Fiebig stages. Second, we found that the plasma viral load associated with the methylation level of *IFI27*. Third, the methylation level of *IFI27* changed concurrent with ART HIV-1 viral suppression and restored to uninfected control levels for those individuals in Fiebig stages II-V that had demethylated their loci in response to HIV-1 viral detection by monocyte cells. Fourth, the level of methylation at *IFI27* significantly associated with gene expression levels of *IFI27* in monocytes. Our data also adds to previous research in the setting of chronic HIV that showed higher gene expression of *IFI27* in people living with HIV-1 above 40,000 copies/ml relative to those than less than 40,000 copies/ml and positive associations with viral load and apoptosis modulation[41–44]. These findings also demonstrate the powerful utility of studying cell type-specific epigenetic patterns in the setting of AHI and highlight methylation patterns of interferon genes as a notable feature to study during the earliest stages of HIV-1 infection and longitudinally following ART.

The role of interferons in HIV-1 disease progression has been a controversial and complicated topic highlighted by Utay and Douek [45]. Our DNA methylation findings in the setting of AHI may provide some additional insight to the HIV-1 interferon debate. Utay and Douek highlighted that the precise timing of type I IFN signaling in AHI is critical to determining clinical outcomes as insufficient IFN signaling during the first week of infection in an SIV infection model results in disease progression[46], and no study has boosted type I IFN signaling during acute HIV infection to examine whether this limits the reservoir. Our findings revealed that DNA methylation states at interferon genes appeared to relate to Fiebig stage and viral sensing by monocyte cells. DNA methylation loss related to the level of gene transcription and viral load in monocytes for interferon genes suggests that demethylation at interferon genes was time-dependent during AHI and that this process would likely relate to innate immune antiviral defense function against HIV-1. Moreover, we observed that early initiation of ART restored DNA methylation of interferon genes in monocytes and thus shut off gene transcription and innate immune antiviral defenses against HIV-1. Given the rapidly advancing field of epigenetic editing and the ability to manipulate DNA methylation levels at specific genomic loci[47,48], we speculate that a cure approach might attempt to simultaneously initiate ART and manipulate the timing of DNA methylation states at interferon genes during AHI, which would lead to maintaining prolonged interferon signaling in innate immune cells. This approach may further limit the HIV-1 reservoir establishment and possibly induce long-term viral control. Epigenetic editing of the host remains understudied in HIV-1 cure.

Our findings suggest that HIV-1 rapidly embeds an indelible epigenetic memory in key innate and adaptive host immune cells during AHI poised for transcriptional dysregulation and immune dysfunction. Recent transcriptomic analyses of the RV144 vaccine trial revealed that in participants of the HIV-1 negative vaccine groups type I interferons that activate the IRF7 antiviral program and type II interferon-stimulated genes were associated with a reduced risk of HIV-1 acquisition [49], suggesting that the regulation of *IRF7* gene is a key mediator of HIV-1 protection and potentially of progression. Our monocyte methylation data in AHI further highlight the function of the *IRF7* gene in HIV-1 infection, provide additional evidence that the *IRF7* plays a key role during the earliest stages of HIV-1 infection, and suggest that DNA methylation plays a key role in regulation *IRF7* gene activity. Of note, previous work showed that IRF7 controls HIV-1 replication in macrophages[24] and monocyte differentiation to macrophages requires IRF7[50], suggesting that IRF7 is a master regulator of both type I interferon-dependent immune responses and dynamics of monocyte-to-macrophage differentiation. We observed that DNA methylation was loss at a regulatory region of the *IRF7* gene in association with AHI. Moreover, the level of DNA methylation at this regulatory region of the *IRF7* gene significantly was associated with the participants CD4 count and CD4/CD8 ratio matching the assayed methylation timepoint. These findings suggest that the level of methylation at *IRF7* in monocytes is a valuable biomarker of immune status and may provide a potential epigenetic target for approaches to increase viral defense prior to and during the earliest stages of infection, potentially leading to viral eradication.

We were struck by the contrast between the extent of DNA methylation changes we observed in monocytes compared to CD4^+^ T lymphocytes. We detected an approximately 7,343% increase in the number of differentially methylated loci in monocytes compared to CD4^+^ T lymphocytes. Moreover, we found that ART had no impact on CD4^+^ T lymphocytes site-specific DNA methylation levels comparing pre-ART to post-ART time points for all AHI participants ranging across Fiebig I to V stages. In contrast, monocytes had significant differences in a small fraction of DML (0.89% of CpGs associated with AHI) following the early initiation of ART during AHI. These findings support the major role of the innate immune system’s viral recognition and immune activation during the early stages of HIV pathogenesis leading to substantial epigenetic remodeling and innate immune cell transcriptional program activation[51]. We did identify 294 DML sites in CD4^+^ T lymphocytes that were associated with AHI at host genes such as *EZR*, *MDM2*, and *PIK3C2A* that play a role in viral activity suggesting that HIV-1 may be modifying CD4^+^ T lymphocytes DNA methylation states to promote viral infection and replication. Additionally, we found that epigenetic age was significantly accelerated in CD4+ T lymphocytes compared to monocytes. We expect that more dramatic changes to DNA methylation states would likely have been observed in infected CD4^+^ T lymphocytes which represent a small fraction of all CD4^+^ T lymphocytes. Yet overall, there is a less dramatic modulation of DNA methylation in CD4^+^ T lymphocytes associated with AHI compared to monocyte cells.

A key feature of specific DNA methylation patterns is that these signatures are cell-type specific. This information has largely been utilized to deconvolute cell type proportion estimations from heterogenous cell populations that have been assayed for DNA methylation profiles[52,53]. Additionally, studies have utilized cell-type specific DNA methylation to develop bioinformatic algorithms intended to correct for cell-type heterogeneity in epigenome-wide association studies[54]. Our study sought to minimize cell-type heterogeneity by profiling purified monocytes and CD4^+^ T lymphocytes. This allowed us to identify both cell type-specific and cell type-independent DML associated with AHI in CD4^+^ T lymphocytes and monocytes. We found that 220 CpGs cell-type independent DML that related to AHI and overlapped in both CD4^+^ T lymphocytes and monocytes. The hypomethylation or hypermethylation state at these DML related to AHI was consistent in both cell types and apparent as early as Fiebig I, suggesting that these regions of the genome may be altered systemically and very early during infection. Monocyte and CD4+ T lymphocyte cell subset differences beyond the resolution of current deconvolution algorithms and defined cell populations are likely involved in a portion of the AHI-related DNA methylation changes. Single cell bisulfite sequencing methods are not feasible across many participants in current clinical cohorts; however, this approach would enhance the resolution at which DNA methylation changes associated with AHI could be linked to specific cells[55]. We have included our uninfected Thai control participants DNA methylation data from CD4+ T cells and monocytes to contribute to efforts to identify marker CpG sites for cell types.

A noted feature of the RV254 AHI cohort is the longitudinal assessment and biospecimen collection of participants following the immediate initiation of ART[18]. For the participants we had assayed DNA methylation levels at study entry during acute HIV infection, we had longitudinal CD4 T cell count data at seven timepoint up to 96 weeks. Hence, we were able to show that the levels of methylation related to the *IRF7* gene in monocyte cells significantly associated with a participant’s CD4 T cell count fold change controlling for baseline CD4 T cell count at all 7 time points following initiation of ART out to 96 weeks. Previous work had shown that initiation of ART during a 4 month time window after HIV-1 infection was associated with an enhanced likelihood of CD4^+^ T lymphocytes counts and CD4^+^ T lymphocytes being preserved with early therapy independent of seroconversion status [56,57]. Our data suggest that personalized epigenetic and functional changes to monocyte cells during acute HIV-1 infection may be an important factor in CD4 T cell recovery in the setting of HIV-1. Indeed previous work has suggested that monocyte-related biomarkers markers relate to poor CD4 T cell recovery [58,59]. The interplay between myeloid cells and CD4^+^ T lymphocytes in HIV-1 and the role of epigenetic mechanisms and implications for risk stratification and therapy should receive further study.

Different factors may drive the persistence of DNA methylation changes in monocytes following ART during AHI. First, there is the possibility that HIV-1 permanently alters DNA methylation in myeloid progenitor cells in the hematopoietic stem cell compartment by viral integration that continually replenish circulating monocytes. Insertion of a provirus has been shown to change the methylation pattern of the host DNA[60]. This concept is controversial despite early research showing that HIV-1 infects purified progenitor cells[61] and occurs in a subset of individuals[62]. Second, the milieu of HIV-1 infection may persist and provide an environment that monocyte cells DNA methylation patterns related to AHI is maintained. This hypothesis would involve HIV-1 persistently inducing a host epigenetic enzyme. Prior work has shown that HIV-1 proteins including Tat, Rev, and Nef have been shown to induce DNMT1 promoter activity which would impact DNA methylation levels[1]. These proteins may also modulate the epigenetic activation or repression of other host genes leading to an epigenetic memory maintained by DNA methylation. Additionally, HIV-1 proteins such as envelope glycoprotein 120 (gp120) persist following ART and have been shown to induce phenotypic changes of monocytes likely involving DNA methylation alterations and transcriptional changes[63]. Whether initiation of a different ART regimens such as one based on inhibition of viral proteins such as a gp120 inhibitor would lead to a more restorative epigenetic state should be evaluated.

Cure strategies for HIV have been challenging and to date only a few people including the Berlin Patient[64] have been cured through arduous non-ART therapeutics. Notably, the few successful HIV cure cases have focused on distinct host genetic features. Epigenetic features of the host have been understudied in HIV-1 cure. We have included the Berlin Patient’s DNA methylation landscape as a reference dataset to inform future studies that seek to examine unique epigenetic host features of individuals who have successfully cleared virus through non-ART therapies. Hence, we believe that charting the epigenetic landscape of distinct immune cells of diverse hosts at different stages of HIV infection will be critical to achieving future cure strategies for HIV-1.

A limitation of this study is the small number of participants identified in the Fiebig I stage of infection precluded a detailed analysis by Fiebig stage. Identification of individuals this early during the course of AHI is challenging. The Fiebig I stage is the earliest stages of acute HIV infection characterized by detectable HIV RNA in plasma but negative detection of HIV-1 in standard diagnostic tests relying on the p24 antigen and HIV-1 antibodies. Despite the limited sample size for individuals in Fiebig I of AHI, our data provide insights into unique epigenetic signatures related to Fiebig I related to the host innate immune response and viral sensing. Previous findings have shown that Fiebig I was associated with significantly lower viral and proviral burden and less immune activation [65]. Moreover, Fiebig I participants in the RV254 cohort were shown to have better CD4^+^ T lymphocytes counts and CD4/CD8 T cell ratios compared to Fiebig II-V individuals. An additional limitation is the lack of longitudinal samples from participants before and after HIV-1 exposure including post-ART timepoints. DNA methylation data from this design would refine the identification of DNA methylation changes due to HIV-1 exposure. Lastly, we also acknowledge that our conclusions were obtained by only studying male AHI participants enrolled in the prospective SEARCH010/RV254 cohort study in Bangkok, Thailand (clinicaltrials.gov NCT00796146). Additional studies are needed to chart sex differences in the DNA methylation landscape of immune cells during AHI and also across HIV-1 clades.

In summary, our epigenetic data provide compelling new information on the earliest host pathogen interaction in key immune cell types during AHI. These findings are relevant to the issue of events during the very early acute HIV-1 period. The cell-type specific epigenetic “hotspots” associated with acute HIV-1 infection we have identified warrant consideration as candidates for epigenome editing approaches in HIV-1 prevention, treatment, and cure. Whether the interindividual epigenetic differences account for individuals’ susceptibility to HIV-1 infection, response to HIV vaccines, and set point following HIV-1 infection also should be a future research direction. Further, discovery of immune-epigenetic predictors of HIV-1 pathogenesis and sequelae open opportunities for novel therapeutic targets.

## Materials and methods

### Ethics statement

The RV254/SEARCH010 study (NCT00796146) was approved by the Institutional Review Boards (IRBs) of Chulalongkorn University, Walter Reed Army Institute of Research (WRAIR), University of California at San Francisco (UCSF), Weill Cornell Medicine, Yale University, University of Hawaii (UH), University of Texas Medical Branch at Galveston, University of Sydney, and Centre Hospitalier de l’Université de Montréal Le Comité d’Ethique de la Recherche. The RV304/SEARCH013 study (NCT01397669) was approved by the IRBs of Chulalongkorn University, Walter Reed Army Institute of Research (WRAIR), University of California at San Francisco (UCSF), and Yale University. The SEARCH011 study (NCT00782808) was approved by the IRBs of Chulalongkorn University, University of California at San Francisco (UCSF), and University of Hawaii (UH). Initiation of ART was voluntary and done as part of enrollment. All participants gave written informed consent for all the studies detailed above.

### Study participants

Specimens from acute HIV-1-infected individuals from the RV254/SEARCH010 study in Bangkok Thailand (clinicaltrials.gov identification NCT00796146) and uninfected healthy individuals from the RV304/SEARCH013 study (clinicaltrials.gov identification NCT01397669) were included in this study. The RV254/SEARCH010 cohort identifies acutely infected individuals by pooled nucleic acid testing using either Roche Amplicor v 1.5 ultrasensitive assay with a lower quantitation limit of 50 copies/ml (Roche Diagnostics, Branchburg, NJ, USA) or Aptima HIV-1 RNA qualitative assay with a lower quantitation limit of 30 copies/ml (Gen-Probe Inc., San Diego, CA, USA) and 4^th^ generation immunoassay that walk in seeking volunteer counseling and testing at The Thai Red Cross Anonymous clinic. Participants identified as HIV-infected are categorized in Fiebig stages I (HIV-1-RNA+, p24 Ag-, HIV-1 IgM-) through Fiebig II-V (HIV-1-RNA+, p24 Ag + or -, HIV-1 IgM–or +). Participants initiated ART within three days of study entry (**S9 Table**). Participants had neuropsychological performance assessed by a neuropsychological battery administered by nurses and research staff who had completed training and qualifying certification tests conducted annually under the direction of a clinical neuropsychologist. The battery included tests that measured fine motor speed and dexterity (non-dominant hand Grooved Pegboard test; GPB; Lafayette Instrument Company, Lafayette, USA), psychomotor speed (Color Trails 1 and Trail Making A[66]) and executive functioning (Color Trails 2[66]). Raw scores were converted to age-, education-, and sex-adjusted standardized z-scores using Thai normative data[67], which were then averaged to create an overall performance score (NPZ).

The study was approved by the institutional review boards (IRBs) of Chulalongkorn University in Thailand and Walter Reed Army Institute of Research in the United States.

Specimens for the Chronic HIV-infected validation cohort were obtained from the SEARCH011 cohort (clinicaltrials.gov identification NCT00782808). SEARCH011 enrolled chronically HIV-infected participants who were ART-naïve and met criteria for initiating therapy according to the Thai Ministry of Health guidelines.

### Purification of monocytes and CD4^+^ T lymphocytes and nucleic acid isolation

Viably cryopreserved peripheral blood mononuclear cells (PBMC) were stored in liquid nitrogen and thawed in RPMI 1640 medium (Hyclone, Logan, Utah, USA) containing 10% heat-inactivated fetal-bovine serum (Hyclone) following established protocols[9]. Cells were stained with propidium iodide (Life Technologies), anti-CD16 Brilliant Violet 421 (Clone 3G8), anti-CD14 BV605 (Clone M5E2), anti-CD3 BV711 (Clone OKT3), anti-CD4 FITC (Clone OKT4), anti-CD7 PE (Clone 6B7), anti-CD11b PerCp/PerCPCY5.5 (Clone ICRF44), anti-CD19 PE-Cy7 (Clone SJ25C1), anti-CD20 PE-Cy7 (Clone 2H7), anti-HLA-DR APC (Clone G46-6). The gating strategy for identification of total monocytes and CD4+ T cells was according to previous reports^11^. Briefly, monocytes were identified by excluding dead cells, lymphocytes (CD3+), and B Cells (CD19+ or CD20+). Monocytes were isolated as HLA-DR+CD11b+ and CD14+ and CD16+ expression. CD4^+^ T lymphocytes were isolated from the CD3+, CD7+, and CD4+ population of cells. DNA and RNA were isolated from sorted total monocytes and CD4^+^ T lymphocytes using the AllPrep DNA/RNA kit (Qiagen) according to the manufacturer’s recommendations for cells. Nucleic acid concentrations were determined using the Qubit DNA Broad Range or RNA Broad Range fluorescence assays (Life Technologies) and Qubit Instrument (Life Technologies).

### Cell-type specific genome-wide DNA methylation profiling

500 ng of DNA per FACS sorted sample (monocytes or CD4+ T cells) were bisulfite converted using the EZ DNA Methylation kit (Zymo Research) according to the manufacturer’s instructions. Bisulfite-converted DNA samples were randomly assigned to a chip well on the Infinium HumanMethylationEPIC BeadChip, amplified, hybridized onto the array, stained, washed, and imaged with the Illumina iScan SQ instrument to obtain raw image intensities at the University of Hawaii Cancer Center Genomics Shared Resource.

### Genome-wide DNA methylation analyses

Raw Methylation EPIC array IDAT intensity data (Data is available at GEO under the accession number GSE180130) was loaded and preprocessed in the R statistical programming language (http://www.r-project.org) using The Chip Analysis Methylation Pipeline (ChAMP, version 2.8.3)[68]. IDAT files were loaded using the champ.load function. All samples passed quality control metrics. Comprehensive filtering was applied to the dataset for probes with detection P-values <0.01, all non-CpG probes, previously published SNP-related probes, multi-hit probes, and probes on sex chromosomes. Methylation beta-values ranging from 0–1 (corresponding to unmethylated to methylated signal intensity) for each sample were normalized using the BMIQ function implemented in the ChAMP pipeline. Differential methylation analysis was conducted on the site level using linear models employed in the limma R package[69]. To identify differentially methylated loci associated with AHI, we compared baseline AHI samples and uninfected control samples. Sites were identified as significant (p<0.05) and filtered for sites based on a biological cut-off with absolute methylation differences greater than 5% (Δβ-value) between groups. DML were annotated using the EPIC array R package annotation IlluminaHumanMethylationEPICanno.ilm10b4.hg19 [70]. CMplot was utilized to generate single track rectangular-Manhattan plots of DML for monocytes and CD4+ T lymphocytes associated with AHI[71]. The EnhancedVolcano R package was utilized to generate volcano plots of DML associated with AHI [72]. Heatmaps were generated using the pheatmap R package [73]. The EWAS toolkit was used for identifying enriched and depleted genomic locations and enriched and depleted histone modifications and chromatin states at DML associated with AHI[74]. Genomic location enrichment analyses included 13 genomic location categories based on the DML location relative to both annotated genes (5’UTR, 1stExon, TSS1500, TSS200, 3’UTR, Intergenic, Body, Island) and CpG islands (N_shelf, N_shore, OpenSea, S-Shelf, S_Shore). Chromatin state and histone modification enrichment used epigenetic data for primary monocytes and T cells from the Roadmap Epigenomics Project[15] that included (DNase, H2A, H3K27ac, H3K27me3, H3K4me1, H3K4me2,H3K4me3, H3K79me2, H3K9ac, H3K9me3, and H4K20me1). The Gene Ontology (GO) and KEGG enrichment analyses of DML used the gometh function in the missMethyl package that controls for the number of probes per gene and multi-gene related probes[75]. For feature selection to identify DNA methylation features associated with a favorable clinical phenotype, we focused on baseline pre-ART DNA methylation associated with AHI in monocytes (22,697 loci) and CD4+ T lymphocytes (294 loci) and included clinical parameters of CD4 count, CD8 count, CD4/CD8 ratio, viral load, and estimated days of exposure. We used models that allowed for up to 2-way interactions among input features and used a 5-fold cross validation with multiple repeats (total of 25 validation trials). Accuracy from the ROC analyses were averaged across the validation trails and served as the final metric of model performance. Classification accuracy (AUC) was also examined using logistic regression to serve as a benchmark comparison to the GBM models.

DNA methylation epigenetic age parameters were calculated using Horvath’s web-based DNAm age calculator tool[19,76]. Epigenetic age acceleration of CD4+ T lymphocytes and monocytes for AHI and control participants was calculated using the difference between calculated Horvath’s DNAmAge and biological age.

### Gene expression

100 ng of total RNA from monocyte cells was utilized for gene expression profiling on AmpliSeq Transcriptome Human Gene Expression Panel for Illumina. Libraries were sequenced on an Illumina NextSeq instrument. We obtained an average of 17.8 million paired end reads per sample with 95.3% of reads aligned over target coding gene regions. Gene expression data was filtered by removing lowly expressed genes and differential gene expression utilized the edgeR package[77].

### Statistical analysis

Plots were drawn using GraphPad Prism software. Differential methylation analyses utilized a linear model implemented in the limma package[78] and did not utilize cell proportion correction methods due to cell-sorted data. Statistical significance of genomic location and chromatin state and histone modification enrichment analyses used a hypergeometic test to calculate a *P* value and odd ratio. Gradient boosted multivariate regression and logistic regression analyses used SciPy[79] to identify DNA methylation features associated with a favorable clinical phenotype and neurocognitive trajectory. Gene expression analyses on count data utilized Empirical Bayes methods and a *P*-adjusted value calculated using the FDR method. Correlations were assessed using the Spearman test.

## Supporting information

S1 Fig AHI-related DNA methylation changes in monocytes and CD4+ T lymphocytes. a. Manhattan plot of differentially methylated loci associated with AHI identified in monocytes and b. CD4+ T lymphocytes. c. Distribution plots of percent of hypo- and hyper-methylated sites and annotated genomic locations of DML in monocytes and d. CD4+ T lymphocytes.
(DOCX)

S2 Fig Unsupervised hierarchical clustering of 220 cell type independent DML. Hypermethylated sites displayed as red and hypo-methylated site displayed as blue. Manhattan distance.
(DOCX)

S3 Fig Epigenetic age acceleration of CD4+ T lymphocytes and monocytes in uninfected, AHI pre-ART, and AHI post-ART participants. a. Bar graph showing mean +SEM epigenetic age acceleration calculated by DNAmAge-Biological Age (Years) in CD4+ T cells (white bar) and monocytes (solid bar) at pre-ART and post-ART timepoints for AHI participants. b. Bar graph showing mean +SEM epigenetic age acceleration calculated by DNAmAge-Biological Age (Years) in CD4+ T cells (white bar) and monocytes (solid bar) in uninfected control participants. c. Bar graph showing mean +SEM epigenetic age acceleration calculated by DNAmAge-Biological Age (Years) in CD4+ T cells (white bar) in uninfected participants, AHI pre-ART, and AHI post-ART. d. Bar graph showing mean +SEM epigenetic age acceleration calculated by DNAmAge-Biological Age (Years) in monocytes (white bar) in uninfected participants, AHI pre-ART, and AHI post-ART. * P< 0.05, **P<0.01. Statistical significance tested using ANOVA with post hoc testing.
(DOCX)

S4 Fig Associations of monocyte transcription, DNA methylation, and clinical immune and viral measures. Correlation plot of AHI participant’s baseline CD4 count, CD4/CD8 ratio, log10 viral load, and site-specific DNA methylation levels related to a. *APOBEC3A*, b. *AIM2*, c. *STAT1*, d. *XRCC4*, e. *PDE4B*, and f. *USP18* genes. Positive correlations displayed in blue and negative correlations in red. Correlation coefficient shown in box.
(DOCX)

S5 Fig Association between CD4 T cell fold change of participants from baseline to post-ART timepoint and DNA methylation level of CpG related to the *IRF7* gene. Fiebig I displayed in red, Fiebig II in blue, and Fiebig III-V in purple color. Left panel shows the relationship to CD4 fold change calculated for AHI participants at Week 12 post-ART and right panel shows CD4 fold change calculated for AHI participants at Week 96 post-ART. Correlative data presented is not corrected for baseline CD4 count.
(DOCX)

S1 Table Genomic Location Enrichment of Top 1000 DML in Monocytes Associated with AHI. (DOCX)

S2 Table Chromatin State of Top 1000 DML in Monocytes Associated with AHI. (DOCX)

S3 Table Gene Ontology Enrichment of Top 1000 DML in Monocytes Associated with AHI. (DOCX)

S4 Table Genomic Location Enrichment of 294 DML in CD4 T Cells Associated with AHI. (DOCX)

S5 Table Chromatin State of Top 294 DML in CD4 T Cells Associated with AHI. (DOCX)

S6 Table Genomic Location Enrichment of 684 DML in monocytes following ART. (DOCX)

S7 Table Chromatin State of 684 DML in Monocytes following ART. (DOCX)

S8 Table Gene Ontology Enrichment of 684 DML in monocytes following ART. (DOCX)

S9 Table AHI Participants ART treatments. (DOCX)

S1 Data Differentially methylated loci associated with AHI in monocytes. (XLSX)

S2 Data Differentially methylated loci associated with AHI in CD4+ T lymphocytes. (XLSX)

S3 Data Overlap of differentially methylated loci associated with AHI in monocytes and CD4+ T lymphocytes. (XLSX)

S4 Data Differentially methylated loci following ART in monocytes. (XLSX)

S5 Data Gene expression of monocytes pre- and post-ART in monocytes. (XLSX)

S6 Data 79 genes that overlapped in DNA methylation and gene expression dataset for monocytes pre-ART and post-ART in AHI. (XLSX)

S7 Data Uninfected control DML between CD4+ T cells and monocytes. (XLSX)
