# Supplementary material for: Abrupt and altered cell-type specific DNA methylation profiles in blood during acute HIV infection persists despite prompt initiation of ART
Source: PLoS Pathog. 2021 Aug 13;17(8):e1009785. doi: 10.1371/journal.ppat.1009785 (PMC8386872; doi:10.1371/journal.ppat.1009785)
Supplement: S1 Fig — a. Manhattan plot of differentially methylated loci associated with AHI identified in monocytes and b. CD4+ T lymphocytes. c. Distribution plots of percent of hypo- and hyper-methylated sites and annotated genomic locations of DML in monocytes and d. CD4+ T lymphocytes. (DOCX) [file ppat.1009785.s001.docx]

**S1 Fig. AHI-related DNA methylation changes in monocytes and CD4+ T lymphocytes. a.** Manhattan plot of differentially methylated loci associated with AHI identified in monocytes and **b.** CD4+ T lymphocytes. **c.** Distribution plots of percent of hypo- and hyper-methylated sites and annotated genomic locations of DML in monocytes and **d.** CD4+ T lymphocytes.

**
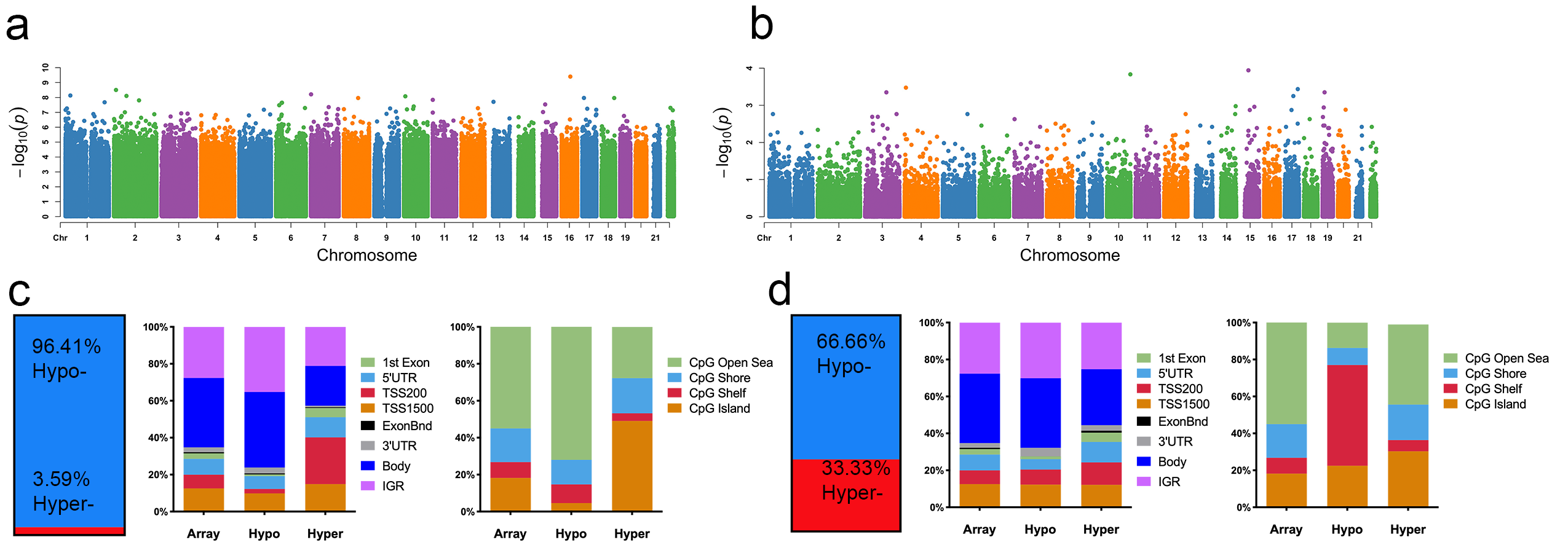
**
