# Supplementary material for: Abrupt and altered cell-type specific DNA methylation profiles in blood during acute HIV infection persists despite prompt initiation of ART
Source: PLoS Pathog. 2021 Aug 13;17(8):e1009785. doi: 10.1371/journal.ppat.1009785 (PMC8386872; doi:10.1371/journal.ppat.1009785)
Supplement: S2 Fig — Hypermethylated sites displayed as red and hypo-methylated site displayed as blue. Manhattan distance. (DOCX) [file ppat.1009785.s002.docx]

**S2 Fig. Unsupervised hierarchical clustering of 220 cell type independent DML.** Hypermethylated sites displayed as red and hypo-methylated site displayed as blue. Manhattan distance.

**
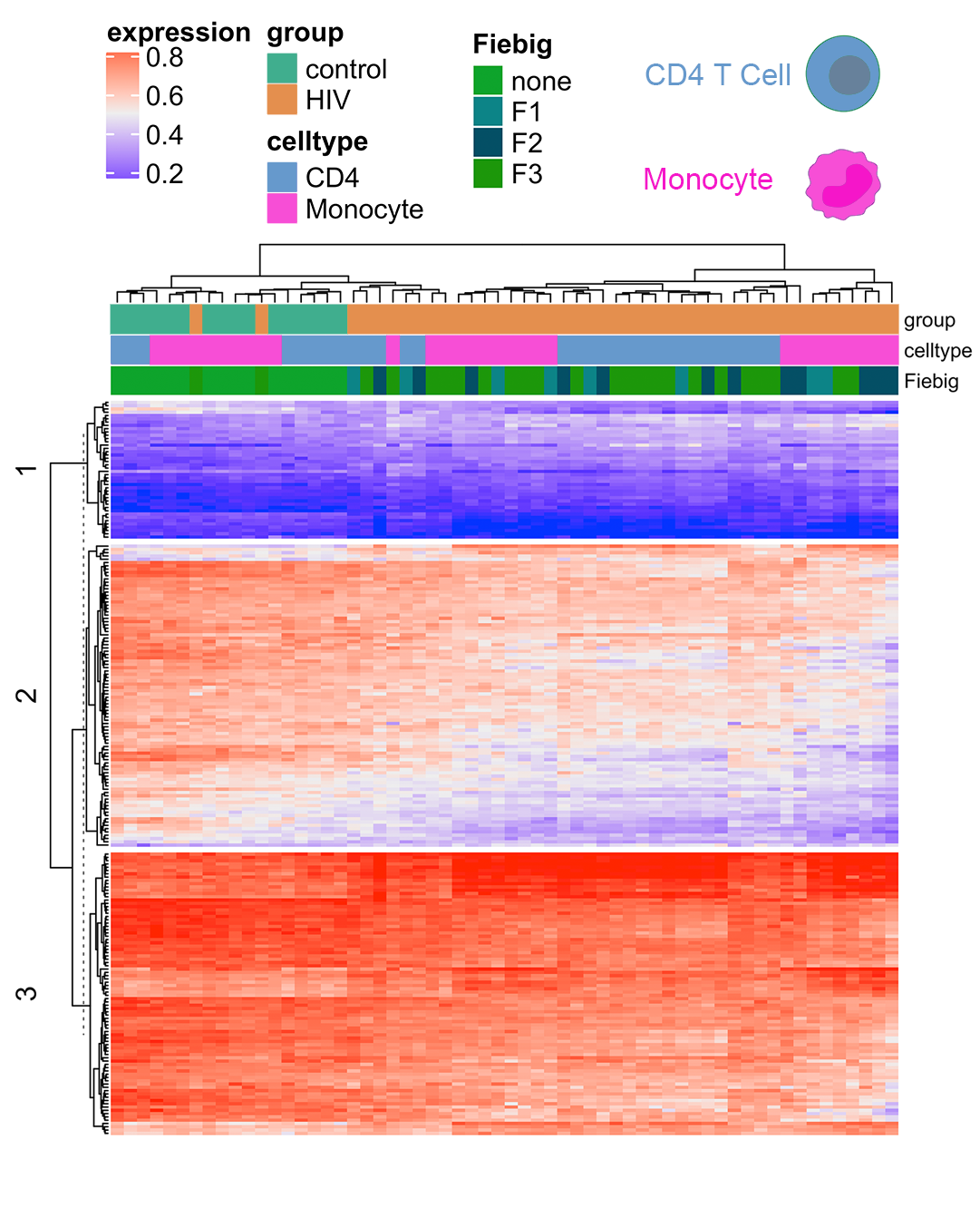
**
