# Supplementary material for: Abrupt and altered cell-type specific DNA methylation profiles in blood during acute HIV infection persists despite prompt initiation of ART
Source: PLoS Pathog. 2021 Aug 13;17(8):e1009785. doi: 10.1371/journal.ppat.1009785 (PMC8386872; doi:10.1371/journal.ppat.1009785)
Supplement: S3 Fig — a. Bar graph showing mean +SEM epigenetic age acceleration calculated by DNAmAge-Biological Age (Years) in CD4+ T cells (white bar) and monocytes (solid bar) at pre-ART and post-ART timepoints for AHI participants. b. Bar graph showing mean +SEM epigenetic age acceleration calculated by DNAmAge-Biological Age (Years) in CD4+ T cells (white bar) and monocytes (solid bar) in uninfected control participants. c. Bar graph showing mean +SEM epigenetic age acceleration calculated by DNAmAge-Biological Age (Years) in CD4+ T cells (white bar) in uninfected participants, AHI pre-ART, and AHI post-ART. d. Bar graph showing mean +SEM epigenetic age acceleration calculated by DNAmAge-Biological Age (Years) in monocytes (white bar) in uninfected participants, AHI pre-ART, and AHI post-ART. * P< 0.05, **P<0.01. Statistical significance tested using ANOVA with post hoc testing. (DOCX) [file ppat.1009785.s003.docx]

**S3 Fig. Epigenetic age acceleration of CD4+ T lymphocytes and monocytes in uninfected, AHI pre-ART, and AHI post-ART participants. a.** Bar graph showing mean +SEM epigenetic age acceleration calculated by DNAmAge-Biological Age (Years) in CD4+ T cells (white bar) and monocytes (solid bar) at pre-ART and post-ART timepoints for AHI participants. **b.** Bar graph showing mean +SEM epigenetic age acceleration calculated by DNAmAge-Biological Age (Years) in CD4+ T cells (white bar) and monocytes (solid bar) in uninfected control participants. **c.** Bar graph showing mean +SEM epigenetic age acceleration calculated by DNAmAge-Biological Age (Years) in CD4+ T cells (white bar) in uninfected participants, AHI pre-ART, and AHI post-ART. **d.** Bar graph showing mean +SEM epigenetic age acceleration calculated by DNAmAge-Biological Age (Years) in monocytes (white bar) in uninfected participants, AHI pre-ART, and AHI post-ART. * P< 0.05, **P<0.01. Statistical significance tested using ANOVA with post hoc testing.

**
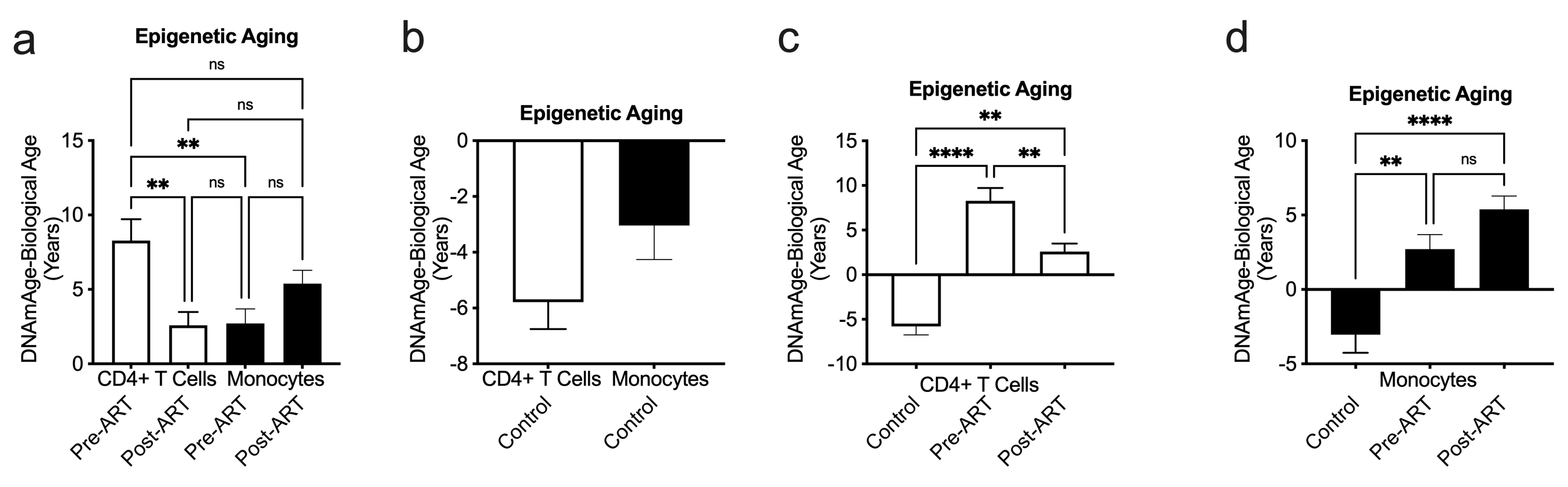
**
