# Supplementary material for: Abrupt and altered cell-type specific DNA methylation profiles in blood during acute HIV infection persists despite prompt initiation of ART
Source: PLoS Pathog. 2021 Aug 13;17(8):e1009785. doi: 10.1371/journal.ppat.1009785 (PMC8386872; doi:10.1371/journal.ppat.1009785)
Supplement: S4 Fig — Correlation plot of AHI participant’s baseline CD4 count, CD4/CD8 ratio, log10 viral load, and site-specific DNA methylation levels related to a. APOBEC3A, b. AIM2, c. STAT1, d. XRCC4, e. PDE4B, and f. USP18 genes. Positive correlations displayed in blue and negative correlations in red. Correlation coefficient shown in box. (DOCX) [file ppat.1009785.s004.docx]

**S4 Fig. Associations of monocyte gene transcription, site-specific DNA methylation, and clinical immune and viral measures.** Correlation plot of AHI participant’s baseline CD4 count, CD4/CD8 ratio, log10 viral load, and site-specific DNA methylation levels related to **a.** *APOBEC3A*, **b.** *AIM2*, **c.** *STAT1*, **d.** *XRCC4*, **e.** *PDE4B,* and **f.** *USP* genes. Positive correlations displayed in blue and negative correlations in red. Correlation coefficient shown in box.

**
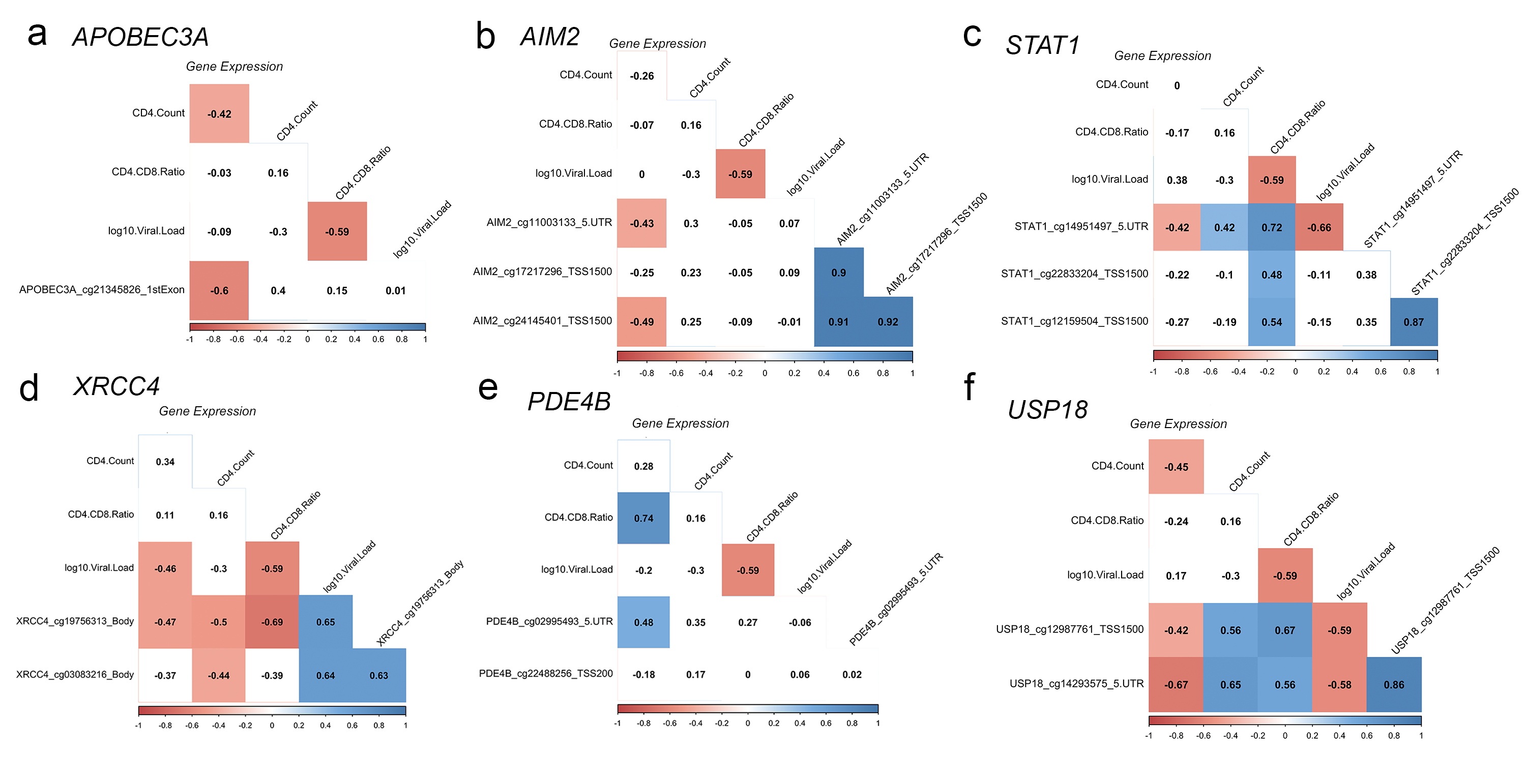
**
