# Supplementary material for: Abrupt and altered cell-type specific DNA methylation profiles in blood during acute HIV infection persists despite prompt initiation of ART
Source: PLoS Pathog. 2021 Aug 13;17(8):e1009785. doi: 10.1371/journal.ppat.1009785 (PMC8386872; doi:10.1371/journal.ppat.1009785)
Supplement: S5 Fig — Fiebig I displayed in red, Fiebig II in blue, and Fiebig III-V in purple color. Left panel shows the relationship to CD4 fold change calculated for AHI participants at Week 12 post-ART and right panel shows CD4 fold change calculated for AHI participants at Week 96 post-ART. Correlative data presented is not corrected for baseline CD4 count. (DOCX) [file ppat.1009785.s005.docx]

**S5 Fig. Association between CD4 T cell fold change of participants from baseline to post-ART timepoint and DNA methylation level of CpG related to the *IRF7* gene.** Fiebig I displayed in red, Fiebig II in blue, and Fiebig III-V in purple color. Left panel shows the relationship to CD4 fold change calculated for AHI participants at Week 12 post-ART and right panel shows CD4 fold change calculated for AHI participants at Week 96 post-ART. Correlative data presented is not corrected for baseline CD4 count.
