# Supplementary material for: Abrupt and altered cell-type specific DNA methylation profiles in blood during acute HIV infection persists despite prompt initiation of ART
Source: PLoS Pathog. 2021 Aug 13;17(8):e1009785. doi: 10.1371/journal.ppat.1009785 (PMC8386872; doi:10.1371/journal.ppat.1009785)
Supplement: S1 Table — (DOCX) [file ppat.1009785.s006.docx]

**S1 Table. Genomic Location Enrichment of Top 1000 DML in Monocytes Associated with AHI.**

| **Genomic Location** | **Odds Ratio** | **p Value** | **Input** | **Background** |
| --- | --- | --- | --- | --- |
| Island | 0.451 | 1.54E-15 | 0.09 | 0.18 |
| TSS200 | 0.349 | 7.31E-13 | 0.035 | 0.094 |
| 1stExon | 0.306 | 3.21E-10 | 0.02 | 0.062 |
| OpenSea | 1.506 | 5.37E-10 | 0.66 | 0.563 |
| TSS1500 | 0.656 | 1.70E-05 | 0.107 | 0.154 |
| Intergenic | 1.312 | 7.39E-05 | 0.326 | 0.269 |
| Body | 1.217 | 2.02E-03 | 0.498 | 0.449 |
| N_Shelf | 1.282 | 7.63E-02 | 0.056 | 0.044 |
| N_Shore | 0.814 | 1.01E-01 | 0.071 | 0.086 |
| S_Shore | 0.822 | 1.21E-01 | 0.069 | 0.083 |
| S_Shelf | 1.23 | 1.44E-01 | 0.054 | 0.044 |
| 3'UTR | 0 | 1.00E+00 | 0 | 0 |
| 5'UTR | 0 | 1.00E+00 | 0 | 0 |
