# Supplementary material for: Abrupt and altered cell-type specific DNA methylation profiles in blood during acute HIV infection persists despite prompt initiation of ART
Source: PLoS Pathog. 2021 Aug 13;17(8):e1009785. doi: 10.1371/journal.ppat.1009785 (PMC8386872; doi:10.1371/journal.ppat.1009785)
Supplement: S2 Table — (DOCX) [file ppat.1009785.s007.docx]

**S2 Table. Chromatin State of Top 1000 DML in Monocytes Associated with AHI.**

| **Cell/Tissue** | **Chromatin State** | **Odds Ratio** | **p value** |
| --- | --- | --- | --- |
| Primary monocytes from peripheral blood | 3_TxFlnk | 4.595 | 5.31E-03 |
| Primary monocytes from peripheral blood | 9_Het | 2.944 | 1.52E-21 |
| Monocytes-CD14+ RO01746 Primary Cells | 9_Het | 2.729 | 4.09E-09 |
| Monocytes-CD14+ RO01746 Primary Cells | 8_ZNF/Rpts | 2.392 | 5.80E-03 |
| Monocytes-CD14+ RO01746 Primary Cells | 5_TxWk | 1.721 | 6.46E-08 |
| Primary monocytes from peripheral blood | 4_Tx | 1.641 | 3.25E-07 |
| Monocytes-CD14+ RO01746 Primary Cells | 4_Tx | 1.569 | 1.49E-06 |
| Primary monocytes from peripheral blood | 5_TxWk | 1.537 | 2.44E-05 |
| Monocytes-CD14+ RO01746 Primary Cells | 2_TssAFlnk | 0.467 | 3.19E-09 |
| Primary monocytes from peripheral blood | 13_ReprPC | 0.427 | 9.77E-09 |
| Monocytes-CD14+ RO01746 Primary Cells | 1_TssA | 0.239 | 3.19E-19 |
| Primary monocytes from peripheral blood | 11_BivFlnk | 0.212 | 8.89E-05 |
| Primary monocytes from peripheral blood | 2_TssAFlnk | 0.207 | 2.42E-14 |
| Primary monocytes from peripheral blood | 1_TssA | 0.19 | 1.73E-22 |
| Monocytes-CD14+ RO01746 Primary Cells | 12_EnhBiv | 0.186 | 1.91E-04 |
| Monocytes-CD14+ RO01746 Primary Cells | 11_BivFlnk | 0.177 | 8.97E-10 |
| Primary monocytes from peripheral blood | 12_EnhBiv | 0.176 | 1.39E-08 |
| Monocytes-CD14+ RO01746 Primary Cells | 10_TssBiv | 0.107 | 2.33E-03 |
