# Supplementary material for: Abrupt and altered cell-type specific DNA methylation profiles in blood during acute HIV infection persists despite prompt initiation of ART
Source: PLoS Pathog. 2021 Aug 13;17(8):e1009785. doi: 10.1371/journal.ppat.1009785 (PMC8386872; doi:10.1371/journal.ppat.1009785)
Supplement: S3 Table — (DOCX) [file ppat.1009785.s008.docx]

**S3 Table. Gene Ontology Enrichment of Top 1000 DML in Monocytes Associated with AHI.**

| **Ontology Type** | **Term** | **p Value** | **FDR** | **DMG** | **Background** |
| --- | --- | --- | --- | --- | --- |
| BP | defense response to virus(GO:0051607) | 8.65E-03 | 1.00E+00 | 11 | 182 |
| BP | sphingolipid biosynthetic process(GO:0030148) | 6.40E-03 | 1.00E+00 | 6 | 43 |
| BP | positive regulation of mitotic cell cycle(GO:0045931) | 6.51E-03 | 1.00E+00 | 5 | 29 |
| BP | neuromuscular process(GO:0050905) | 5.64E-04 | 1.00E+00 | 5 | 19 |
| BP | histone phosphorylation(GO:0016572) | 8.74E-03 | 1.00E+00 | 4 | 12 |
| BP | lysosomal transport(GO:0007041) | 5.73E-03 | 1.00E+00 | 4 | 16 |
| BP | cellular zinc ion homeostasis(GO:0006882) | 6.65E-03 | 1.00E+00 | 3 | 11 |
| MF | zinc ion transmembrane transporter activity(GO:0005385) | 6.52E-03 | 1.00E+00 | 3 | 11 |
| BP | negative regulation of transcription elongation from RNA polymerase II promoter(GO:0034244) | 3.59E-03 | 1.00E+00 | 3 | 10 |
| BP | response to interferon-alpha(GO:0035455) | 2.34E-03 | 1.00E+00 | 3 | 10 |
| CC | GARP complex(GO:0000938) | 2.29E-03 | 1.00E+00 | 3 | 5 |
| BP | positive regulation of interferon-gamma biosynthetic process(GO:0045078) | 2.26E-03 | 1.00E+00 | 3 | 12 |
| BP | adenosine to inosine editing(GO:0006382) | 9.46E-03 | 1.00E+00 | 2 | 4 |
| BP | negative regulation of inflammatory response to antigenic stimulus(GO:0002862) | 9.44E-03 | 1.00E+00 | 2 | 6 |
| BP | phosphatidylglycerol biosynthetic process(GO:0006655) | 9.42E-03 | 1.00E+00 | 2 | 3 |
| CC | serine C-palmitoyltransferase complex(GO:0017059) | 7.88E-03 | 1.00E+00 | 2 | 5 |
| MF | serine C-palmitoyltransferase activity(GO:0004758) | 7.88E-03 | 1.00E+00 | 2 | 5 |
| BP | regulation of fertilization(GO:0080154) | 6.30E-03 | 1.00E+00 | 2 | 5 |
| BP | type I pneumocyte differentiation(GO:0060509) | 5.34E-03 | 1.00E+00 | 2 | 3 |
| BP | regulation of monocyte differentiation(GO:0045655) | 2.96E-03 | 1.00E+00 | 2 | 2 |
| BP | DNA demethylation of male pronucleus(GO:0044727) | 2.44E-03 | 1.00E+00 | 2 | 2 |
