# Supplementary material for: Abrupt and altered cell-type specific DNA methylation profiles in blood during acute HIV infection persists despite prompt initiation of ART
Source: PLoS Pathog. 2021 Aug 13;17(8):e1009785. doi: 10.1371/journal.ppat.1009785 (PMC8386872; doi:10.1371/journal.ppat.1009785)
Supplement: S4 Table — (DOCX) [file ppat.1009785.s009.docx]

**S4 Table. Genomic Location Enrichment of 294 DML in CD4 T Cells Associated with AHI.**

| **Genomic Location** | **Odds Ratio** | **p Value** | **Input** | **Background** |
| --- | --- | --- | --- | --- |
| Island | 1.482 | 4.82E-03 | 0.245 | 0.18 |
| TSS200 | 1.429 | 4.52E-02 | 0.129 | 0.094 |
| N_Shelf | 1.409 | 1.55E-01 | 0.061 | 0.044 |
| 1stExon | 1.275 | 2.76E-01 | 0.078 | 0.062 |
| TSS1500 | 1.123 | 4.67E-01 | 0.17 | 0.154 |
| S_Shore | 1.121 | 5.26E-01 | 0.092 | 0.083 |
| Body | 1.013 | 9.07E-01 | 0.452 | 0.449 |
| Intergenic | 0.88 | 3.58E-01 | 0.245 | 0.269 |
| OpenSea | 0.807 | 6.84E-02 | 0.51 | 0.563 |
| S_Shelf | 0.758 | 4.78E-01 | 0.034 | 0.044 |
| N_Shore | 0.654 | 9.50E-02 | 0.058 | 0.086 |
| 3'UTR | 0 | 1.00E+00 | 0 | 0 |
| 5'UTR | 0 | 1.00E+00 | 0 | 0 |
