# Supplementary material for: Abrupt and altered cell-type specific DNA methylation profiles in blood during acute HIV infection persists despite prompt initiation of ART
Source: PLoS Pathog. 2021 Aug 13;17(8):e1009785. doi: 10.1371/journal.ppat.1009785 (PMC8386872; doi:10.1371/journal.ppat.1009785)
Supplement: S5 Table — (DOCX) [file ppat.1009785.s010.docx]

**S5 Table. Chromatin State of Top 294 DML in CD4 T Cells Associated with AHI.**

| **Cell/Tissue** | **Chromatin State** | **Odds Ratio** | **p value** |
| --- | --- | --- | --- |
| Primary T cells from peripheral blood | 8_ZNF/Rpts | 7.05 | 9.56E-03 |
| Primary T helper memory cells from peripheral blood 1 | 4_Tx | 2.602 | 1.61E-03 |
| Primary T helper cells from peripheral blood | 1_TssA | 1.87 | 1.95E-04 |
| Primary T helper cells PMA-I stimulated | 4_Tx | 1.813 | 2.22E-03 |
| Primary T cells effector/memory enriched from peripheral blood | 4_Tx | 1.768 | 5.10E-03 |
| Primary T helper naive cells from peripheral blood 2 | 4_Tx | 1.738 | 4.00E-03 |
| Primary T helper cells from peripheral blood | 4_Tx | 1.67 | 6.54E-03 |
| Primary T helper cells PMA-I stimulated | 1_TssA | 1.595 | 3.71E-03 |
| Primary T regulatory cells from peripheral blood | 1_TssA | 1.587 | 9.27E-03 |
| Primary T helper naive cells from peripheral blood 1 | 1_TssA | 1.51 | 7.85E-03 |
| Primary T helper memory cells from peripheral blood 2 | 1_TssA | 1.505 | 9.03E-03 |
| Primary T helper memory cells from peripheral blood 2 | 7_Enh | 0.458 | 4.49E-03 |
| Primary T helper cells PMA-I stimulated | 7_Enh | 0.44 | 9.73E-03 |
| Primary T helper 17 cells PMA-I stimulated | 7_Enh | 0.41 | 8.05E-03 |
| Primary T helper naive cells from peripheral blood 2 | 7_Enh | 0.4 | 6.15E-03 |
| Primary T helper naive cells from peripheral blood 1 | 14_ReprPCWk | 0.352 | 1.06E-03 |
| Primary T helper naive cells from peripheral blood 1 | 7_Enh | 0.286 | 4.21E-04 |
| Primary T helper cells from peripheral blood | 7_Enh | 0.277 | 2.15E-04 |
| Primary T regulatory cells from peripheral blood | 13_ReprPC | 0.241 | 6.75E-04 |
| Primary T helper cells from peripheral blood | 13_ReprPC | 0.177 | 1.86E-03 |
| Primary T helper 17 cells PMA-I stimulated | 13_ReprPC | 0.094 | 6.54E-04 |
|  |  |  |  |
