# Supplementary material for: Abrupt and altered cell-type specific DNA methylation profiles in blood during acute HIV infection persists despite prompt initiation of ART
Source: PLoS Pathog. 2021 Aug 13;17(8):e1009785. doi: 10.1371/journal.ppat.1009785 (PMC8386872; doi:10.1371/journal.ppat.1009785)
Supplement: S6 Table — (DOCX) [file ppat.1009785.s011.docx]

**S6 Table. Genomic Location Enrichment of 684 DML in monocytes following ART.**

| **Genomic Location** | **Odds Ratio** | **p Value** | **Input** | **Background** |
| --- | --- | --- | --- | --- |
| OpenSea | 2.172 | 6.06E-21 | 0.737 | 0.563 |
| Body | 1.363 | 5.28E-05 | 0.526 | 0.449 |
| S_Shelf | 1.303 | 1.13E-01 | 0.057 | 0.044 |
| N_Shelf | 1.026 | 8.52E-01 | 0.045 | 0.044 |
| Intergenic | 0.983 | 8.63E-01 | 0.266 | 0.269 |
| N_Shore | 0.95 | 7.85E-01 | 0.082 | 0.086 |
| TSS1500 | 0.819 | 8.07E-02 | 0.13 | 0.154 |
| S_Shore | 0.725 | 4.37E-02 | 0.061 | 0.083 |
| 1stExon | 0.337 | 1.09E-06 | 0.022 | 0.062 |
| TSS200 | 0.186 | 1.85E-15 | 0.019 | 0.094 |
| Island | 0.082 | 7.53E-42 | 0.018 | 0.18 |
| 3'UTR | 0 | 1.00E+00 | 0 | 0 |
| 5'UTR | 0 | 1.00E+00 | 0 | 0 |
