# Supplementary material for: Abrupt and altered cell-type specific DNA methylation profiles in blood during acute HIV infection persists despite prompt initiation of ART
Source: PLoS Pathog. 2021 Aug 13;17(8):e1009785. doi: 10.1371/journal.ppat.1009785 (PMC8386872; doi:10.1371/journal.ppat.1009785)
Supplement: S7 Table — (DOCX) [file ppat.1009785.s012.docx]

**S7 Table. Chromatin State of 684 DML in Monocytes following ART.**

| **Cell/Tissue** | **Chromatin State** | **Odds Ratio** | **p value** |
| --- | --- | --- | --- |
| Primary monocytes from peripheral blood | 7_Enh | 10.412 | 2.01E-176 |
| Monocytes-CD14+ RO01746 Primary Cells | 3_TxFlnk | 8.923 | 2.50E-12 |
| Monocytes-CD14+ RO01746 Primary Cells | 7_Enh | 8.46 | 1.51E-104 |
| Primary monocytes from peripheral blood | 3_TxFlnk | 8.103 | 1.27E-04 |
| Primary monocytes from peripheral blood | 6_EnhG | 7.237 | 4.17E-24 |
| Monocytes-CD14+ RO01746 Primary Cells | 6_EnhG | 4.648 | 2.18E-10 |
| Monocytes-CD14+ RO01746 Primary Cells | 2_TssAFlnk | 3.26 | 8.00E-36 |
| Monocytes-CD14+ RO01746 Primary Cells | 4_Tx | 0.616 | 1.14E-03 |
| Primary monocytes from peripheral blood | 4_Tx | 0.609 | 1.66E-03 |
| Monocytes-CD14+ RO01746 Primary Cells | 15_Quies | 0.332 | 8.88E-27 |
| Primary monocytes from peripheral blood | 15_Quies | 0.3 | 3.25E-31 |
| Monocytes-CD14+ RO01746 Primary Cells | 14_ReprPCWk | 0.213 | 1.14E-12 |
| Monocytes-CD14+ RO01746 Primary Cells | 9_Het | 0.162 | 7.43E-04 |
| Primary monocytes from peripheral blood | 14_ReprPCWk | 0.118 | 2.41E-14 |
| Monocytes-CD14+ RO01746 Primary Cells | 11_BivFlnk | 0.11 | 2.05E-08 |
| Monocytes-CD14+ RO01746 Primary Cells | 13_ReprPC | 0.104 | 2.58E-19 |
| Monocytes-CD14+ RO01746 Primary Cells | 1_TssA | 0.096 | 2.05E-21 |
| Primary monocytes from peripheral blood | 11_BivFlnk | 0.077 | 8.69E-05 |
| Primary monocytes from peripheral blood | 9_Het | 0.066 | 1.23E-10 |
| Primary monocytes from peripheral blood | 13_ReprPC | 0.065 | 4.03E-20 |
| Primary monocytes from peripheral blood | 1_TssA | 0.027 | 2.70E-27 |
