# Supplementary material for: Abrupt and altered cell-type specific DNA methylation profiles in blood during acute HIV infection persists despite prompt initiation of ART
Source: PLoS Pathog. 2021 Aug 13;17(8):e1009785. doi: 10.1371/journal.ppat.1009785 (PMC8386872; doi:10.1371/journal.ppat.1009785)
Supplement: S8 Table — (DOCX) [file ppat.1009785.s013.docx]

**S8 Table. Gene Ontology Enrichment of 684 DML in monocytes following ART.**

| **Term** | **p Value** | **FDR** | **DMG** | **Background** |
| --- | --- | --- | --- | --- |
| cytosol(GO:0005829) | 1.05E-03 | 9.18E-01 | 148 | 4871 |
| cytoplasm(GO:0005737) | 9.95E-03 | 1.00E+00 | 100 | 3433 |
| identical protein binding(GO:0042802) | 5.67E-03 | 1.00E+00 | 39 | 1041 |
| defense response to virus(GO:0051607) | 4.44E-13 | 7.76E-09 | 23 | 182 |
| innate immune response(GO:0045087) | 1.06E-05 | 2.66E-02 | 21 | 386 |
| viral process(GO:0016032) | 3.21E-03 | 1.00E+00 | 18 | 366 |
| type I interferon signaling pathway(GO:0060337) | 1.93E-11 | 1.69E-07 | 15 | 67 |
| response to virus(GO:0009615) | 4.06E-07 | 1.77E-03 | 13 | 103 |
| negative regulation of gene expression(GO:0010629) | 4.66E-03 | 1.00E+00 | 12 | 232 |
| nuclear chromatin(GO:0000790) | 2.16E-03 | 1.00E+00 | 12 | 167 |
| interferon-gamma-mediated signaling pathway(GO:0060333) | 2.54E-05 | 5.54E-02 | 11 | 72 |
| negative regulation of viral genome replication(GO:0045071) | 9.23E-10 | 5.37E-06 | 11 | 40 |
| double-stranded DNA binding(GO:0003690) | 7.51E-06 | 2.19E-02 | 10 | 72 |
| PML body(GO:0016605) | 6.37E-03 | 1.00E+00 | 9 | 99 |
| tumor necrosis factor-mediated signaling pathway(GO:0033209) | 8.82E-03 | 1.00E+00 | 7 | 115 |
| double-strand break repair(GO:0006302) | 7.81E-03 | 1.00E+00 | 5 | 54 |
| tumor necrosis factor receptor binding(GO:0005164) | 3.01E-04 | 3.76E-01 | 5 | 30 |
| positive regulation of defense response to virus by host(GO:0002230) | 9.67E-05 | 1.88E-01 | 5 | 26 |
| cellular response to interferon-beta(GO:0035458) | 4.80E-06 | 1.68E-02 | 5 | 19 |
| positive regulation of cytokinesis(GO:0032467) | 5.93E-03 | 1.00E+00 | 4 | 36 |
