# Supplementary material for: Abrupt and altered cell-type specific DNA methylation profiles in blood during acute HIV infection persists despite prompt initiation of ART
Source: PLoS Pathog. 2021 Aug 13;17(8):e1009785. doi: 10.1371/journal.ppat.1009785 (PMC8386872; doi:10.1371/journal.ppat.1009785)
Supplement: S9 Table — (DOCX) [file ppat.1009785.s014.docx]

**S9 Table. AHI Participant ART Treatments**

| **Participant ID** | **ART Regimen** |
| --- | --- |
| *AHI_1* | TDF/FTC/EFV |
| *AHI_2* | TDF/FTC/EFV/MVC/RAL |
| *AHI_3* | TDF/FTC/EFV |
| *AHI_4* | TDF/FTC/EFV/MVC/RAL |
| *AHI_5* | TDF/FTC/EFV |
| *AHI_6* | TDF/FTC/EFV/MVC/RAL |
| *AHI_7* | TDF/FTC/EFV/MVC/RAL |
| *AHI_8* | TDF/FTC/EFV/MVC/RAL |
| *AHI_9* | TDF/3TC/EFV/MVC/RAL |
| *AHI_10* | TDF/3TC/EFV |
| *AHI_11* | TDF/3TC/EFV |
| *AHI_12* | TDF/3TC/EFV |
| *AHI_13* | TDF/3TC/EFV |
| *AHI_14* | TDF/3TC/EFV |
| *AHI_15* | TDF/3TC/EFV |
| *AHI_16* | AZT/3TC/LPV/RTV |
| *AHI_17* | TDF/3TC/EFV |
| *AHI_18* | TDF/3TC/EFV |
| *AHI_19* | TDF/3TC/EFV |
| *AHI_20* | TDF/3TC/EFV |
| *AHI_21* | TDF/3TC/EFV |
| *AHI_22* | TDF/3TC/EFV |
